# Preventive action of benztropine on platinum-induced peripheral neuropathies and tumor growth

**DOI:** 10.1186/s40478-019-0657-y

**Published:** 2019-01-18

**Authors:** Olivier Cerles, Tânia Cristina Gonçalves, Sandrine Chouzenoux, Evelyne Benoit, Alain Schmitt, Nathaniel Edward Bennett Saidu, Niloufar Kavian, Christiane Chéreau, Camille Gobeaux, Bernard Weill, Romain Coriat, Carole Nicco, Frédéric Batteux

**Affiliations:** 1 Department “Development, Reproduction and Cancer”, Institut Cochin, Paris Descartes University, Sorbonne Paris City, INSERM U1016, Paris, France; 2 Molecular engineering of proteins unit (SIMOPRO), CEA of Saclay, and Paris-Saclay Institute of Neuroscience (Neuro-PSI), UMR CNRS 9197, Paris-Saclay University, Gif-sur-Yvette, France; 3 Sanofi R & D, Integrated Drug Discovery – High Content Biology, Vitry-sur-Seine, France; 4 Plateforme imagerie : microscopie électronique, Institut Cochin, INSERM U1016, Paris, France; 5 Department of Immunology, Cochin Teaching Hospital, AP-HP, 27, rue du faubourg Saint-Jacques, F75014 Paris, France; 6 Service de diagnostic biologique automatisé, Cochin Teaching Hospital, Paris, France; 7 Department of Gastroenterology, Cochin Teaching Hospital, Paris Descartes University, Paris, France

**Keywords:** Benztropine, Oxaliplatin, Peripheral neuropathies, Muscarinic receptors, Myelin

## Abstract

The endogenous cholinergic system plays a key role in neuronal cells, by suppressing neurite outgrowth and myelination and, in some cancer cells, favoring tumor growth. Platinum compounds are widely used as part of first line conventional cancer chemotherapy; their efficacy is however limited by peripheral neuropathy as a major side-effect. In a multiple sclerosis mouse model, benztropine, that also acts as an anti-histamine and a dopamine re-uptake inhibitor, induced the differentiation of oligodendrocytes through M1 and M3 muscarinic receptors and enhanced re-myelination. We have evaluated whether benztropine can increase anti-tumoral efficacy of oxaliplatin, while preventing its neurotoxicity.

We showed that benztropine improves acute and chronic clinical symptoms of oxaliplatin-induced peripheral neuropathies in mice. Sensory alterations detected by electrophysiology in oxaliplatin-treated mice were consistent with a decreased nerve conduction velocity and membrane hyperexcitability due to alterations in the density and/or functioning of both sodium and potassium channels, confirmed by action potential analysis from ex-vivo cultures of mouse dorsal root ganglion sensory neurons using whole-cell patch-clamp. These alterations were all prevented by benztropine. In oxaliplatin-treated mice, MBP expression, confocal and electronic microscopy of the sciatic nerves revealed a demyelination and confirmed the alteration of the myelinated axons morphology when compared to animals injected with oxaliplatin plus benztropine. Benztropine also prevented the decrease in neuronal density in the paws of mice injected with oxaliplatin. The neuroprotection conferred by benztropine against chemotherapeutic drugs was associated with a lower expression of inflammatory cytokines and extended to diabetic-induced peripheral neuropathy in mice.

Mice receiving benztropine alone presented a lower tumor growth when compared to untreated animals and synergized the anti-tumoral effect of oxaliplatin, a phenomenon explained at least in part by benztropine-induced ROS imbalance in tumor cells.

This report shows that blocking muscarinic receptors with benztropine prevents peripheral neuropathies and increases the therapeutic index of oxaliplatin. These results can be rapidly transposable to patients as benztropine is currently indicated in Parkinson’s disease in the United States.

**Electronic supplementary material:**

The online version of this article (10.1186/s40478-019-0657-y) contains supplementary material, which is available to authorized users.

## Introduction

Chemotherapy-induced peripheral neuropathy (CIPN) is a severe and long lasting side effect caused by diverse anticancer agents that damage sensory and/or motor nerves. CIPN occurs in 30–70% of patients treated with specific categories of anticancer agents [72]. Symptoms of CIPN include numbness, pain, burning, tingling, heat/cold hyperalgesia, and mechanical allodynia, as well as reduced motor function. CIPN commonly presents a “stocking-glove” distribution with the most distal portions of the limb exhibiting the greatest deficits [9]. These symptoms usually begin after multiple doses of the chemotherapeutic agents, and progress as treatment continues. After the treatments end, they can resolve in a short time period, or persist as an after-effect of cancer therapy.

Oxaliplatin-associated CIPN is the most frequent cause of interruption of an otherwise successful therapy [34]. About 12 to 18% of patients experience the highest grade 3 CIPN evaluated on the National Cancer Institute’s Common Terminology Criteria for Adverse Events (NCI-CTCAE) scale [4, 21], when persistent neuropathy graded more than 2 usually results in complete discontinuation of the chemotherapy.

Oxaliplatin is a platinum compound indicated in solid tumor cancers [38, 87], which induces peripheral neuropathies of two forms: an acute but reversible form [54] which manifests within hours following the first infusion and a chronic form that occurs following cumulative high doses of the drug and is non-reversible [66]. Several clinical manifestations characterize this condition, but hallmark symptoms include persistent distal chronic paresthesia, allodynia and thermoalgesia. Indeed, the severity of the pain endured by some patients may require treatment arrest, rendering this neuropathy a limiting-factor for this chemotherapy.

The mechanisms behind the neurotoxicity of chemotherapy are complex and are not fully understood [6, 15]. Neurotoxicity may be due to chemotherapy interactions with DNA, mitochondrial perturbations, ion channel dysregulations, reactive oxygen balance modifications, kinases and/or glutamatergic neurotransmission dysfunction. The lesions can occur at the level of the dorsal root ganglia (DRG), at the level of the sensory neurons, associated with defects of axonal transport related to the microtubules or to degeneration of the axon. They may also affect Schwann cells by inducing dedifferentiation and mitochondrial [37] and satellite cell [13] dysfunction. The complexity of the involved mechanisms partly explains the lack of effective treatments to prevent CIPN.

Diabetic neuropathies (DN) are similar in their anatomo-pathological features to oxaliplatin-induced peripheral neuropathies, as they involve demyelination, axonal swelling and reduced cutaneous nerve fiber density [36, 74]. A progressive development with sensory loss, pain and autonomic dysfunction are common symptoms. Pathologically, DN is characterized by interrelated metabolic abnormalities with insulin deficiency and hyperglycemia as the initiating culprits. The neuropathy accompanying type 2 DM (insulin resistance) and type 1 DM (insulin deficiency) appears to differ, the former showing a milder axonal involvement and segmental myelin breakdown, whereas the latter shows a more severe axonal atrophy and axonal loss. The disorder does not only involve somatic peripheral nerves but also autonomic and central nerve tracts. Today no successful therapy exists for DN.

In both CIPN [13] and DN [25], myelin-forming Schwann cells of the peripheral nervous system are crucial for the proper function and maintenance of peripheral nerves. Schwann cells wrap around axons and thereby provide insulation, acceleration of electric signal propagation, and axonal protection and maintenance. Schwann cells are the main effectors for regeneration in a variety of peripheral neuropathic conditions [31, 32, 51], including inherited, inflammatory, toxic (e.g. CIPN), and DN, as well as traumatic injuries to peripheral nerve fibers. Due to their high differentiation plasticity, these cells respond to injury and disease by myelin sheath degradation, dedifferentiation into an immature Schwann cell-like phenotype. In doing so, they can support and promote axonal regrowth and target tissue innervation.

Based on our current knowledge, it is evident that Schwann cells play a key role in the pathogenesis of various inflammatory, metabolic and hereditary polyneuropathies [31, 51]. Over the last two decades, fundamental progress has been made in understanding the molecular basis of Schwann cell biology. These advances have led to the development of new treatment strategies that aim to improve the protective and regenerative properties of Schwann cells in peripheral nerve disorders. The neuroprotective potential of a molecule which improves clinical symptoms of experimental autoimmune encephalomyelitis in mice by remyelinating neurons has recently been reported [22]. Benztropine, an inhibitor of acetylcholine (ACh) muscarinic M1 and M3 receptors (mAChR), has been shown to promote oligodendrocyte precursor cells differentiation while allowing greater axonal remyelination than molecules currently prescribed to multiple sclerosis patients [22]. Interestingly, M3 and M1 mAChR are also expressed on peripheral myelin forming Schwann cells but their roles in the myelination process remain unclear. In a multiple sclerosis mouse model, benztropine induced the differentiation of oligodendrocytes through M1 and M3 muscarinic receptors and enhanced re-myelination [22]. In addition to its anti-cholinergic effects, benztropine also produces anti-histaminic effects [58], inhibits the re-uptake of terminal nerve dopamine [2] and is an allosteric antagonist of the human D2 dopamine receptor (Pubchem BioAssay: AID 485344 and Drugbank.ca: DB00245).

However, targeting a pathway to prevent CIPN must be considered with caution, since this additional treatments may directly or indirectly favor tumor growth or dissemination. Interestingly, in the case of muscarinic receptor blockade, several studies have highlighted the potential of targeting M3 muscarinic receptors to prevent tumor growth and metastases [17, 19, 77]. Furthermore, ACh-induced tumorigenesis has been reported in several organs that are commonly treated with oxaliplatin [17, 91]**.** The differential distribution between mAChR subtypes could allow the specific targeting of tumor cells**.**

These observations of potential for both preventing neurotoxicity by promoting axonal protection as well as increasing the efficacy profile of neurotoxic chemotherapies, prompted us to investigate the effects of the mAChR inhibitor, benztropine, associated with oxaliplatin, in vitro and in vivo on CIPN, DN and tumor growth.

## Materials and methods

### Cell culture and treatments

CT26 (mouse colon carcinoma) cells, purchased from the ATCC (CRL-2638), were grown in Dulbecco’s Modified Eagle Medium (Gibco, Sigma-Aldrich) with 10% FBS (Gibco, Sigma-Aldrich), penicillin/streptomycin, and _L_-glutamine supplementation. N2a (mouse brain neuroblastoma) cells, purchased from the ATCC (CCL-131), were grown in Eagle Minimum Essential Medium supplemented with 10% FBS, penicillin/streptomycin, 2% sodium pyruvate, 1% ciprofloxacin, non-essential amino acids, and _L_-glutamine supplementation.

### Animal models and treatments

Five-week-old male BALB/cJRj mice were purchased from Janvier Laboratory (Le Genest Saint Isle, France). All mice were housed in ventilated cages (*n* = 7 to 10 animals per cage) with sterile food and water ad libitum and were exposed to a standard light cycle of 12 h on and 12 h off. All experiments were performed in accordance with European and French institutional guidelines (Directive 2010/63/EU, Ethics committee CEEA 34 – APAFIS authorization #8394).

### Model of oxaliplatin-induced peripheral neuropathies

Both models of oxaliplatin-induced peripheral neuropathies, namely, the acute form and the chronic form were induced by protocols with timeframes that were analogous to the clinical protocols which results in the induction of either of these forms in patients, and were previously used in mice.

The acute form of oxaliplatin peripheral neuropathy was induced in mice (*n* = 7 per group) by daily low doses intraperitoneal injections of oxaliplatin (3 mg/kg diluted in phosphate-buffered saline (PBS 1X)) for 5 days followed by 5 days of rest and another 5 days of oxaliplatin at the same dosage, as previously described [16, 18, 79]. Benztropine (10 mg/kg diluted in PBS) was administered as daily intraperitoneal injections 6 h after oxaliplatin injection, 5 days a week during the 2 cycles of oxaliplatin injection and the rest period.

The chronic form of oxaliplatin peripheral neuropathy mice (*n* = 25 per group for all the experiments) was induced by intraperitoneal injections of oxaliplatin (10 mg/kg diluted in PBS) once a week each Mondays for 6–8 weeks as previously described [16, 18, 89]. Benztropine (10 mg/kg diluted in PBS) was administered as daily intraperitoneal injections 6 h after oxaliplatin injection, 5 days a week for 6–8 weeks.

### Model of streptozotocin-induced diabetic peripheral neuropathies

Poor survival over the course of preliminary tests was observed with a 3-h food withdrawal before and after a single dose of 200 mg/kg of Streptozotocin (STZ) [39]. This dose was therefore lowered to 180 mg/kg (dissolved in 10 mmol/L sodium citrate buffer, pH 4.5) with 3-h food fasting before and after the injection (*n* = 8 per group). STZ was diluted to allow a final volume of 0.1 ml/10 g body weight for intraperitoneal injections. One week after STZ injection, diabetes was confirmed by blood glucose levels using a Cobas 8000 modular analyzer (Roche) in blood samples taken under isoflurane 2,5% (Isovet, #ISO005, Centravet, Plancoët, France) from mice retro-orbital sinus. Mice with blood glucose levels ≥12 mM were considered diabetic and included in the study [81].

### Behavioral studies

Prior to baseline measurements for every behavioral test, mice underwent 2 weeks of acclimation sessions with the specific training protocols to familiarize them with the experimental environments and testing procedures, without probing with plastic fibers nor temperature setting. Before each behavioral test, mice were allowed to acclimate to the experimental room for 10 min in their home cages. Mice were only tested once on any given test day to avoid any possible stress, anesthetic or tissue damage effects that could result from repeated exposure to the cold surface.

### In vivo cold hyperalgesia

Mice from the acute model of oxaliplatin-induced peripheral neuropathies were submitted to weekly tests for cold hyperalgesia. Mice were put on a cold/hot plate (Ugo Basile, Comerio, Italy) set at the temperature of 2 °C ± 0.2 °C [16, 18, 79]. The total number of brisk lifts from either hind paw or jumps, considered as a painful response to cold, was counted simultaneously by two observers to ensure accuracy and averaged for the two observers’ counts. A maximal cut-off time of 5 min was imposed to prevent tissue damage. Results are expressed as the mean ± SEM of the observers’ counts, with 7 different mice under each condition.

### In vivo cold hypoesthesia

Mice from the chronic model of oxaliplatin-induced peripheral neuropathies were submitted to weekly tests for cold hypoesthesia which was evaluated using temperature settings previously described [16, 18]. Briefly, mice were put on the cold/hot plate set at 4 °C ± 0.2 °C for 5 min. Similar, to the test assessing cold hyperalgesia, the total number of brisk lifts from either hind paw or jumps was counted simultaneously by two observers to ensure accuracy and averaged for the two observers’ counts. Results are expressed as the mean ± SEM of the observers’ counts, with 8 different mice under each condition.

### In vivo tactile hypoesthesia

Mice from both the chronic oxaliplatin-induced and the diabetic models underwent weekly tests for mechanical allodynia with the von Frey method [53], a standardized protocol assessing tactile sensitivity [61]. A set of 20 monofilaments based on the Semmes Weinstein monofilament set was used (Model: Bio-VF-M, Bioseb, USA/Canada). The Semmes Weinstein set of monofilaments provides an approximate logarithmic scale of actual force, and a linear scale of perceived intensity. Briefly, mice were put on a mesh grid, an enclosed by a clear plexiglass barrier with a top cover and left to calm down for 5 min. After the settling phase, mice are motionless allowing the experimenter to touch their hind paws with a flexible plastic fiber of a fixed diameter. The fiber is pressed through the mesh against the plantar surface at a right angle. The force of application increases as long as the investigator pushes the probe and until the fiber bends. The scale of force used ranged from 0.008 to 1.400 g. The threshold to perception of probing was asserted to the movement of pulling back of either hind paw of 8 different mice under each condition.

### In vivo hot hyperesthesia

Mice from the diabetic model of peripheral neuropathies underwent a weekly assessment of thermal hyperalgesia according to a previously published study protocol [81]. The same equipment as for cold sensitivity was used for this experiment. The plate was set at 55 °C ± 0.2 °C. Latency to hind paw licking or jumping was recorded simultaneously by two observers with a timer and time to response was averaged for the two counts, for 8 different mice under each condition. To prevent tissue damage a cutoff time of 30 s was implemented.

### In vivo electrophysiological exploration of neuromuscular and sensory excitability

The sensory and/or neuromuscular excitability was assessed in vivo on mice under isoflurane (AErrane®) anesthesia by minimally invasive electrophysiological methods using the Qtrac© software (Prof. H. Bostock, Institute of Neurology, London, England), as previously described [16]. Briefly, an anaesthetized mouse (*n* = 10) was placed on a heating pad to maintain body temperature (from 36.02 ± 0.06 to 36.13 ± 0.04, *n* = 85) throughout the experiments to avoid non-specific modifications of excitability variables.

For neuromuscular excitability exploration, electrical stimulation was delivered to the sciatic motor nerve by means of surface electrodes, and the compound muscle action potential (CMAP) was recorded using needle electrodes inserted into the plantar muscle. Each mouse was systematically submitted to one session of excitability measurements (TRONDE protocol), which consisted of five different excitability tests performed together: (C0) The stimulus-response relationship (i.e. the CMAP amplitude as a function of the intensity of a 1-ms stimulation) evaluated notably both the CMAP maximal amplitude and the stimulation intensity that had to be applied to evoke a CMAP of 50% maximal amplitude, giving information on the global neuromuscular excitability state. (C1) The current-threshold relationship evaluated the threshold changes at the end of 200-ms conditioning subthreshold depolarizing and hyperpolarizing currents ranging from 50 to 100% thresholds, giving information on axonal accommodation capacities to depolarizations and hyperpolarizations. (C2) The strength-duration relationship (i.e.*,* the intensity in relation to the duration of a stimulus necessary to evoke a given amplitude of CMAP) evaluated the minimal intensity of infinitely long duration stimulation necessary to evoke a CMAP (rheobase) and the intensity duration of twice the rheobase stimulation necessary to evoke a CMAP (chronaxis), giving information on the axonal resting potential at the nodal membrane. (C3) The threshold electrotonus (i.e.*,* the threshold changes during and after 100-ms conditioning subthreshold depolarizing and hyperpolarizing currents applied at ±40% thresholds) evaluated the electrotonic changes in membrane potential, giving also information on axonal accommodation capacities to depolarizations and hyperpolarizations. (C4) The recovery cycle (i.e.*,* the excitability changes that occur following a CMAP) evaluated the refractory periods (during which membrane excitability is either nil or markedly decreased) followed by the supernormal and late subnormal periods (during which membrane excitability is increased and decreased, respectively). As a whole, more than 30 variables were determined from these excitability tests and analyzed. Most of them provide specific and complementary information on the density and functional state of ion channels, receptors and pumps, as well as on the passive membrane properties of the neuromuscular system [45, 46].

For sensory excitability exploration, the compound nerve action potential (CNAP) was recorded using needle electrodes inserted into the base of the tail, in response to stimulation of the caudal nerve applied at the distal part of the tail by means of surface electrodes. Each mouse was systematically and only submitted to the first session of excitability measurements (TRONDE protocol) to establish the stimulus-response relationship (i.e.*,* the CNAP amplitude as a function of the intensity of a 1-ms stimulation) and thus, evaluate notably the CNAP maximal amplitude, the stimulation intensity that had to be applied to evoke a CNAP of 50% maximal amplitude and the latency measured from stimulation onset to peak amplitude, giving information on the global sensory excitability state.

### In vitro electrophysiological exploration of sensory excitability

In vitro electrophysiological exploration of sensory excitability was performed by recording the action potential from primary cultures of mouse dorsal root ganglia (DRG) sensory neurons, using the patch-clamp technique. After being removed from the spinal cord of euthanized adult female Swiss mice (10–12 weeks of age and 28–32 g body weight, purchased from Janvier Elevage and housed at the CEA animal facility), DRG were placed in iced-Ham’s F-12 medium (Sigma-Aldrich, Saint-Quentin Fallavier, France) and enzymatically dissociated with collagenase type IA (2 mg/mL; Sigma-Aldrich) and dispase (5 mg/mL; Gibco, Thermo Fisher Scientific, Villebon-sur-Yvette, France). Neurons were then plated on 12-mm glass coverslips placed in a 24-wells plate coated with 10 μg/mL of poly-D-lysine and 100 μg/mL of murin laminin (Sigma-Aldrich). The cells were maintained in culture at 37 °C (in 95% air and 5% CO_2_) in Neurobasal A medium (Gibco) containing horse serum (5%; Gibco), penicillin/streptomycin (47.64 U/mL; Gibco), nerve growth factor (83.33 ng/mL; Sigma-Aldrich), N2 supplement (3.18x; Gibco), Dulbecco’s PBS (1X) w/o CaCl_2_ and MgCl_2_ (1.68%; Gibco), bovine serum albumin (16.83 μg/mL; Sigma-Aldrich), corticosteron (214.85 nM; Sigma-Aldrich), T3 hormone (56.06 nM; Sigma-Aldrich) and L-glutamine (1.90 mM; Sigma-Aldrich). Cytosine β-D-arabinofuranoside (2 μM; Sigma-Aldrich) was added to the culture medium, 24 h later, to stop astrocyte proliferation. Experiments were carried out within 2 to 6 days after cell plating. The day of their use, the neurons plated on coverslips were transferred, for a minimum of 30 min at 37 °C prior to patch-clamp recordings, in 35-mm Petri dishes filled with a standard physiological medium of the following composition (in mM): NaCl 134, KCl 3, CaCl_2_ 1, MgCl_2_ 1, D-glucose 20, and HEPES 20 (pH 7.35, adjusted with NaOH), and then in the recording bath filled with the standard physiological medium.

Whole-cell patch-clamp experiments were performed under current-clamp condition, by using a MultiClamp 700B integrating patch-clamp amplifier and the pClamp10.6 software (Molecular Devices, Sunnyvale, CA, USA), as previously described [70]. The signals, acquired at a 4-kHz sample rate, were filtered at 2 kHz with a low-pass Bessel filter and digitized with the aid of a computer equipped with an analog-to-digital converter (Digidata-1440A model; Molecular Devices). The patch-clamp pipettes were filled with a medium composed of (in mM): KCl 134, NaCl 10, MgCl_2_ 2, EGTA 2, ATP-Na_2_ 4, and HEPES 20 (pH 7.32, adjusted with KOH), and had 2.71 ± 0.25 MΩ resistance (*n* = 18) in the standard physiological medium. A fast superfusion system allowed changing the solution [standard physiological medium without or with oxaliplatin (25–50 μM) alone or oxaliplatin (25–50 μM) plus benztropine (10 μM)] around the recorded cell within a few seconds. The experiments were carried out at constant room temperature (22 °C). Action potentials were elicited, at a frequency of 0.5 Hz, by 100-ms current test-pulses of − 0.2 to 1 nA (in 0.1-nA increments) applied 200 ms after 200-ms current pre-pulses of − 0.1 nA (to check the membrane passive properties of neurons, mainly membrane capacitance).

### Ex vivo confocal microscopy morphological study of sciatic nerves

The experiments were carried out on single myelinated axons isolated from the sciatic nerves of euthanized mice, as previously detailed [16]. Briefly, sciatic nerve sections (*n* = 4 mice in each group) of about 2 cm in length were removed from their sheaths, dissected, and fixed for 1 h in PBS 1X with 2% paraformaldehyde, then rinsed three times with PBS. Sciatic nerves were deposited on microscope slides, myelinated axons were gently teased apart from the main trunk, and preparations were kept at − 20 °C until use. Just before the experiments, sciatic nerves were rehydrated for about 1 h with a standard physiological solution containing (in mM): NaCl 154, KCl 5, CaCl_2_ 2, MgCl_2_ 1, glucose 11, and HEPES 5 (pH 7.4, adjusted with NaOH. Preparations were then exposed for 30 min to the fluorescent dye FM1–43 (Molecular Probes) dissolved in a standard physiological solution to stain the plasma membranes of the myelinated axons, and washed with dye-free solution before imaging. A Zeiss LSM 510 META (Carl Zeiss) multiphoton scanning confocal microscope, mounted on an upright microscope and controlled with the manufacturer’s software and workstation, was used for optical sectioning of myelinated axons and subsequent 3D high-resolution digital reconstruction of their structure. Images were collected using a 63x oil-immersion objective with a 1.40 numerical aperture (Zeiss Plan-Apochromat); following excitation of FM1–43 with the 488 nm wavelength line of an Argon ion laser, and then digitized at 12-bit resolution into a 512 × 512 pixel array. Images were then analyzed using the ImageJ software (National Institutes of Health - NIH). Quantification of morphometric parameters of myelinated axons were performed by measuring the internodal diameter, nodal diameter (D) and nodal length (L). Assuming the simplest geometry in which a node of Ranvier approaches a cylinder, the nodal volume (V) was then determined as V = μL(D/2)^2^.

### Ex vivo electronic microscopy morphological study of sciatic nerves

Mice were anesthetized by intraperitoneal injections of 100 mg/kg ketamine and 10 mg/kg xylazine and then intracardially perfused with first, 0.1 M PBS, pH 7.4, for 8 min and then 4% paraformaldehyde, 2.5% glutaraldehyde, and 0.1 M PBS, pH 7.4. Mice were euthanized after complete rigidity of the lower limbs and liver (endpoint of the perfusion). Tissues were dissected 24 h later and immersed in the fixative solution at 4 °C for 72 h, washed in PBS, post-fixed in 2% osmium tetroxide, dehydrated in graded ethanol, and embedded in epoxy resin. Ultrathin sections (50–90 nm) were cut on an ultramicrotome (8800 Ultrotome III; LKB Bromma) and collected on 300-mesh nickel grids. Staining was performed on drops of 4% aqueous uranyl acetate, followed by Reynolds lead citrate. Ultrastructural analyses were performed in a JEOL JEM-1011 electron microscope and digitalized with DigitalMicrograph software. G-ratios and axon diameters mice (*n* = 2 to 3 mice in each group) were calculated with the ImageJ software (National Institutes of Health) and the plugin *g*-ratio version 3.2 (Plug-in and source code available online at http://gratio.efil.de) according to previously published material [30]. Diameters were calculated from enclosed areas, considering first, the diameter of the axon without the myelin and dividing it by the diameter of the axon plus the myelin sheaths surrounding it. The plugin allows for semi-automated analysis of randomly selected fibers with both diameters being considered and automatically processed through the algorithm to calculate the g-ratio. A minimum of 500 randomly selected axons were analyzed per experimental group, with at least 3 mice per group.

### Ex vivo study of cutaneous nerve fiber density

Sections of skin from the hind paws of 8 mice per group were preserved in buffered 4% formol (VWR Chemicals, Labonord SAS, France) and immersed in successive baths of increasing concentrations of ethanol (50, 70, 90, 100%). Samples were then immersed in histosol before being fixed in paraffin, kept at 4 °C and cut into 6 μm-thick slices. Samples were then unwaxed in three successive baths of histosol for 3 min and rehydrated in ethanol baths for 1 min each, starting with two baths of pure ethanol followed with baths of 90, 70 and 30% ethanol and two baths of H_2_O. Slides were then washed in PBS for 2 min before being immersed in citrate buffer at 95 °C for 10 min. Samples were washed in 3 baths of PBS of 5 min each. Samples were then permeabilized (PBS, 0.25% Triton X-100) for 10 min before being rinsed in PBS in 3 baths of 5 min each and immersed in a blocking solution (PBS, 10% goat serum, 1% BSA) for at least 180 min. Samples were further incubated at 4 °C overnight in the primary antibody (Abcam, ab10404, rabbit polyclonal to PGP9.5**,** 1:1000) before being rinsed in PBS in 3 baths of 5 min each before incubating in the secondary antibody (Sigma, F9887, anti-rabbit IgG FITC-conjugate, 1:1000) at room temperature in the dark. Prior to mounting (Thermo Scientific, Shandon Immuno-Mount), slides were washed with PBS in 3 baths of 5 min each. Images were collected using a Nikon Eclipse 80i microscope with a Nikon PlanFluor 100x/1.30 oil-immersion DIC H/N2 objective.

### Ex vivo study of myelin protein content in sciatic nerves

Nerves were extracted in lysis buffer containing a cocktail of proteinase and phosphatase inhibitors using a glass homogenizer. Protein lysate samples (40 g) were resolved on 10% SDS-PAGE gels and transferred by electrophoresis to nitrocellulose membranes. The membranes were blocked for non-specific binding sites at room temperature in TBS buffer containing 0.1% Tween 20 and 5% nonfat dried milk for 1 h. The membranes were then incubated overnight at 4 °C with a primary antibody against MBP (1:500; Merck). Subsequently, the membranes were incubated with an anti-rabbit IgG, horseradish peroxidase linked whole antibody from donkey (1:1000; GE Healthcare Life Sciences NA934, Little Chalfont, UK) and subjected to ECL reagent treatment. After film exposure, all membranes were washed and then incubated with a 1:50,000 dilution of mouse monoclonal anti-actin-peroxidase antibody (Sigma Aldrich; Saint Louis, Missouri, USA) as previously described [52].The images were captured using a CCD camera (LAS3000 from Fujifilm), and the bands were quantified using MultiGauge software from Fujifilm.

### Ex vivo study of systemic inflammatory markers

Blood samples were taken under isoflurane from the retro-orbital sinus immediately before sacrifice (*n* = 8 per group), centrifuged at 10.000 rpm and 2 °C. Sera were diluted (1:4) in ELISA/ELISPOT diluent 1X before being distributed on ELISA 96-well plates specific of IL-6 and Tumor necrosis factor alpha (TNF-α; Mouse IL-6 ELISA Ready-SET-Go!® and Mouse TNF ELISA Ready-SET-Go!® - eBioscience, San Diego, CA, USA). Concentrations were calculated from a standard curve according to the manufacturer’s protocol.

### In vivo study of antitumor activity of benztropine upon association with chemotherapy

A total of 1.10^6^ viable CT26 cells, as determined by trypan blue staining, and resuspended in DMEM were injected subcutaneously into the back of the mice. When tumors reached a mean size of 200 to 500 mm^3^, animals were randomized and received a single weekly injection of either oxaliplatin (10 mg/kg) or vehicle. Concomitantly, mice received vehicle or benztropine (10 mg/kg) every day. Following randomization, tumor size was measured with a numeric caliper twice a week for 3 weeks. In order to comply with ethical guidelines, tumor growth experiments were stopped 3 weeks after the first oxaliplatin injection. Tumor volume was calculated as follows: *TV* (mm^3^) = (*L* x *W*^2^)/2, where *L* is the longest and *W* the shortest radius of the tumor in millimeters. Results are expressed as mean ± SD of tumor volumes (*n* = 7 in each group).

### Viability and ROS assays

All cells (2 × 10^4^ per well) were seeded in 96-well plates (Sigma-Aldrich, Saint-Quentin Fallavier France) and incubated for 24 h with 7.5 to 30 μM of benztropine (Sigma-Aldrich, Saint-Quentin Fallavier France) and treated with 0 to 100 μM of oxaliplatin (Accord Healthcare Limited, Lille, France). Cell viability was assessed by a crystal violet assay, and results are expressed as the mean percentage of viable cells ± SEM versus cells not exposed to oxaliplatin (100% viability). Cellular production of ROS and reduced glutathione (GSH) were assessed by spectrofluorimetry with 2′,7′-dichlorodihydrofluorescein diacetate (H2DCFDA, #6883 Sigma-Aldrich, Saint-Quentin Fallavier France) and monochlorobimane (#69899 Sigma-Aldrich), respectively [49].

### Statistical analysis

Statistical analysis was performed using GraphPad Prism 5. Artwork, was also created using GraphPad Prism 5, except for electrophysiological study artwork which was created using the Qtrac© software. Differences between values were tested using the unpaired Student’s *t*-test, two-way ANOVA, or the nonparametric Mann-Whitney *U* test, depending on the equality of variances estimated using Lilliefors test. They were considered significant when *p* < 0.05, *p* values being denoted as follows: *p < 0.05, **p < 0.01, ***p < 0.001, ****p < 0.0001; NS: non-significant.

## Results

### In vivo effects of oxaliplatin and benztropine on mouse cold hyperalgesia

**Fig. 1:**
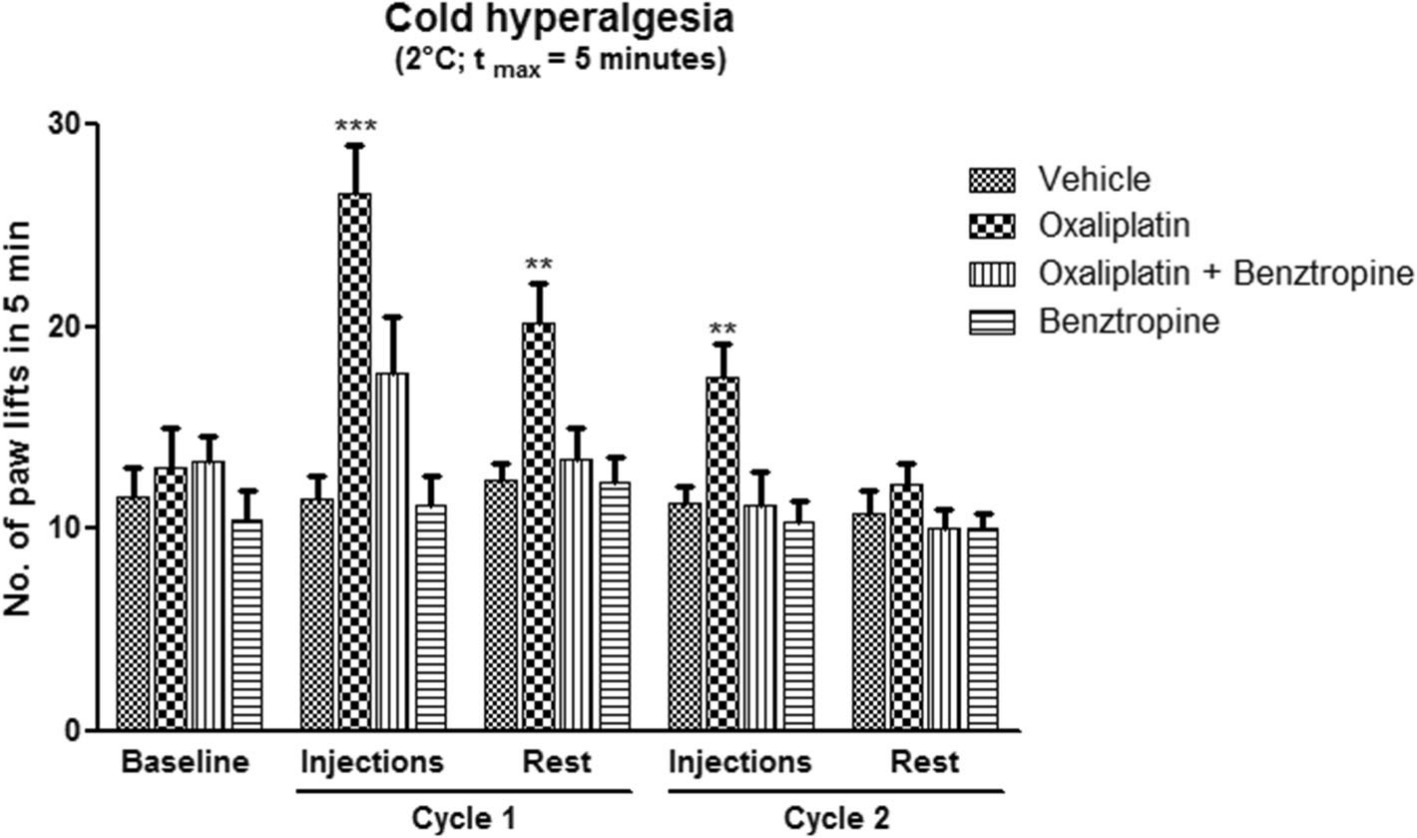
In vivo effects of oxaliplatin and benztropine on acute oxaliplatin peripheral neuropathy. Evaluation of hyperalgesia required two 5-day cycles of daily oxaliplatin (3 mg/kg). Control mice received either oxaliplatin or vehicle alone. Cold hyperalgesia was evaluated using a cold plate set at + 2 °C. Data are expressed as means ± SEM of 7 different mice under each condition. *p < 0.05, **p < 0.01, ***p < 0.001 versus vehicle

To determine whether benztropine could abrogate oxaliplatin-induced peripheral neuropathy, cold hyperalgesia was evaluated in acute oxaliplatin-exposed mice treated or not with benztropine. After oxaliplatin cycle 1, the mean number of brisk lifts was over twice as high in oxaliplatin-treated mice than in animals injected with vehicle alone (26.57 ± 2.45 with oxaliplatin versus 11.43 ± 1.13 with vehicle, *p* = 0.0001). The same changes were observed after the second injection of oxaliplatin (17.43 ± 1.70 with oxaliplatin versus 11.29 ± 0.84 with vehicle, *p* = 0.0071). Association with benztropine rescued this hyperalgesia from the first injection of oxaliplatin (17.71 ± 2.76 with oxaliplatin plus benztropine versus 11.43 ± 1.13 with vehicle, *p* = 0.0566) (Fig. 1).

### In vivo effects of oxaliplatin and benztropine on mouse cold and tactile hypoesthesia

**Fig. 2:**
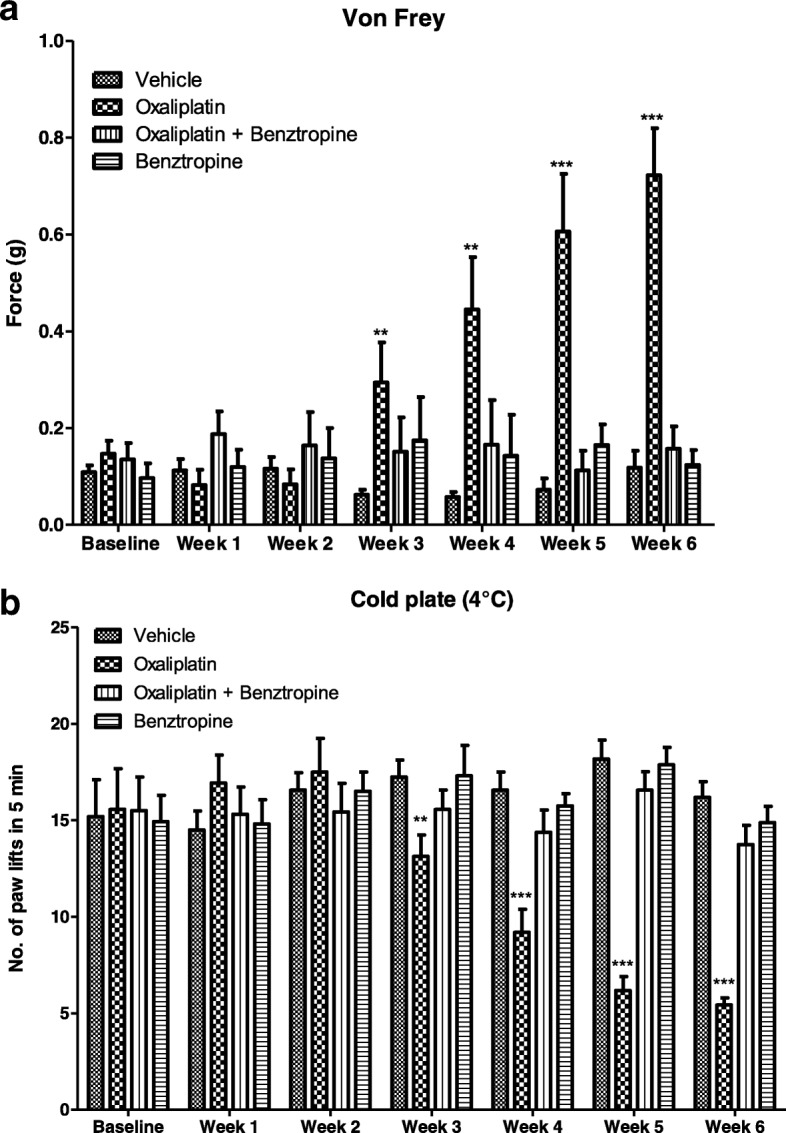
In vivo effects of oxaliplatin and benztropine on chronic oxaliplatin peripheral neuropathy. **a** von Frey test and **b** cold-plate hypoesthesia test. Experimental mice received oxaliplatin (10 mg/kg) weekly and benztropine (10 mg/kg) daily for 6 weeks. Control mice received either oxaliplatin or vehicle alone. Both the von Frey and the cold-plate tests were performed on a weekly basis. Data are expressed as means ± SEM of 8 different mice under each condition. *p < 0.05, **p < 0.01, ***p < 0.001 versus vehicle

In addition to acute cold hyperalgesia, after a long term treatment, patients treated with oxaliplatin also suffer from permanent pathological thermal and tactile perception at their extremities. Submitted to the chronic form oxaliplatin-induced peripheral neuropathy, mice injected with 10 mg/kg oxaliplatin developed diminished tactile perception from week 3 of treatment (0.2419 ± 0.0687 with oxaliplatin versus 0.0625 ± 0.0107 with vehicle, *p* = 0.0151) with a peak tactile hypoesthesia observed at the end of the testing period (at week 6, 0.7225 ± 0.0973 with oxaliplatin versus 0.1188 ± 0.0348 with vehicle, *p* < 0.0001). Mice treated with benztropine associated with the chemotherapy did not display these symptoms of altered tactile hypoesthesia at week 3 (0.1515 ± 0.0704 with oxaliplatin plus benztropine versus 0.0625 ± 0.0107 with vehicle, *p* = 0.2212) nor at any time point during the experiment (at week 6, 0.1575 ± 0.0458 with oxaliplatin plus benztropine versus 0.1188 ± 0.0348 with vehicle, *p* = 0.5054) (Fig. 2a). Mice injected with 10 mg/kg oxaliplatin developed reduced cold hypoesthesia from week 3 of treatment (13.13 ± 1.11 with oxaliplatin versus 17.25 ± 0.87 with vehicle, *p* = 0.0066). The most severe cold hypoesthesia in oxaliplatin-treated mice was observed at the end of the testing period (at week 6, 5.44 ± 0.36 with oxaliplatin versus 16.19 ± 0.81 with vehicle, *p* < 0.0001). Mice treated with benztropine associated with the chemotherapy did not display these symptoms of altered cold hypoesthesia at week 3 (15.56 ± 1.00 with oxaliplatin plus benztropine versus 17.25 ± 0.87 with vehicle, *p* = 0.2145) nor at any time point during the experiment (at week 6, 13.75 ± 0.99 with oxaliplatin plus benztropine versus 16.19 ± 0.81 with vehicle, *p* = 0.0659) (Fig. 2b).

### In vivo effects of benztropine on diabetic mouse tactile hypoesthesia and hot hyperesthesia

**Fig. 3:**
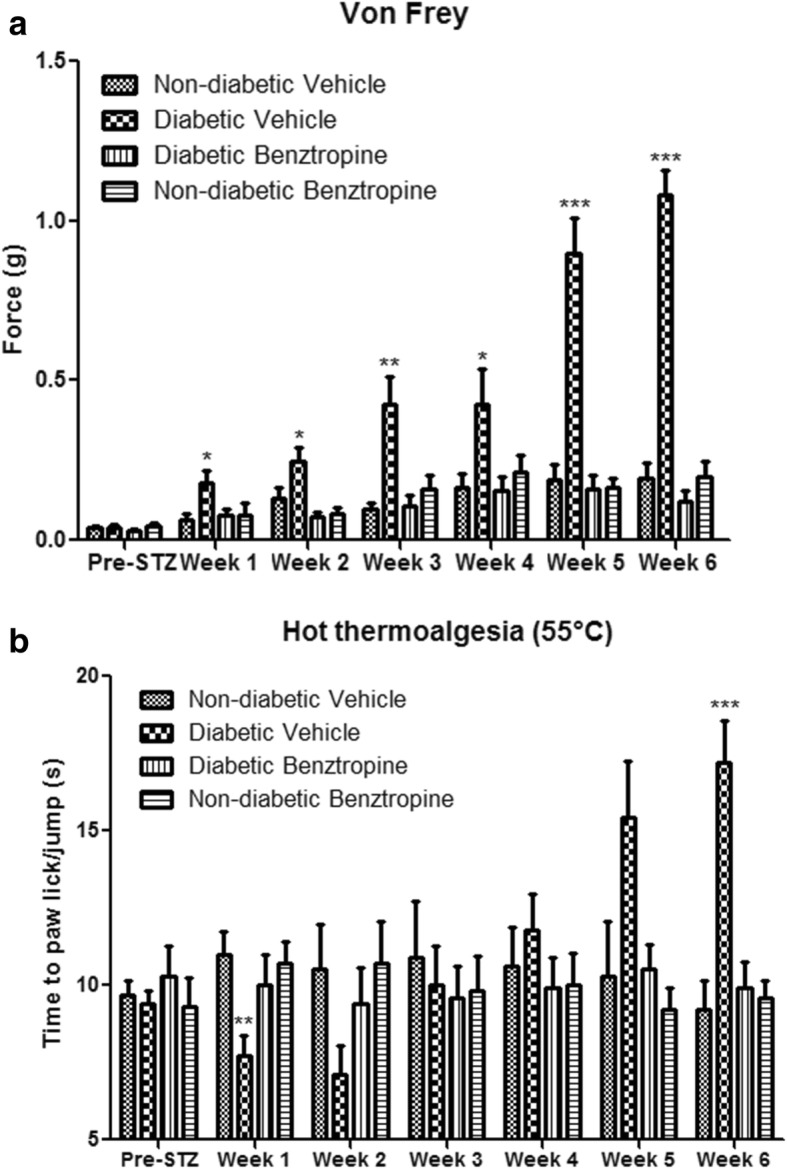
In vivo effects of oxaliplatin and benztropine on diabetes-induced peripheral neuropathies. **a** von Frey test and **b** hot-plate test. Experimental diabetic mice received benztropine (10 mg/kg) daily for 6 weeks. Control mice received either benztropine or vehicle alone. Both, the von Frey as well as the hot-plate tests were performed on a weekly basis. Data are expressed as means ± SEM of 8 different mice under each condition. *p < 0.05, **p < 0.01, ***p < 0.001 versus vehicle

Since peripheral neuropathies can result from causes other than oxaliplatin infusions, we sought to investigate whether benztropine could alleviate painful symptoms brought about by other etiologies. Mice models of streptozotocin (STZ)-induced diabetes are robust and well-documented [81]. These mice develop peripheral neuropathies similar to those witnessed in diabetic patients. Diabetic mice developed diminished tactile perception from week 1 (0.1780 ± 0.0398 in diabetic mice versus 0.0636 ± 0.0173 in non-diabetic mice, *p* = 0.0168). Peak tactile hypoesthesia in diabetic mice was observed at the end of the testing period (week 6, 1.0800 ± 0.0800 in diabetic mice versus 0.1950 ± 0.0481 in non-diabetic mice, *p* < 0.0001). Diabetic mice treated with benztropine did not display these symptoms of altered tactile hypoesthesia at week 1 (0.0768 ± 0.0191 in benztropine-treated diabetic mice versus 0.0636 ± 0.0173 in non-diabetic mice, *p* = 0.6153) nor at any time point during the experiment (at week 6, 0.1200 ± 0.0355 in benztropine-treated diabetic mice versus 0.1950 ± 0.0481 in non-diabetic mice, *p* = 0.2253) (Fig. 3a). Diabetic mice developed transient heat hyperalgesia from week 1 (7.70 ± 0.68 in diabetic mice versus 11.00 ± 0.75 in non-diabetic mice, *p* = 0.0043). Diabetic mice treated with benztropine did not display these symptoms of exacerbated pain to the hot plate at week 1 (10.00 ± 0.97 in benztropine-treated diabetic mice versus 11.00 ± 0.75 in non-diabetic mice, *p* = 0.4232) or symptoms of hot hypoalgesia at week 6 (9.90 ± 0.85 in benztropine-treated diabetic mice versus 9.20 ± 0.93 in non-diabetic mice, *p* = 0.5849), at which it peaked in diabetic mice (17.20 ± 1.36 in diabetic mice versus 9.20 ± 0.93 in non-diabetic mice, *p* = 0.0001) (Fig. 3b). Benztropine corrected both thermal perception abnormalities, namely transient hot hyperalgesia and persistent hot hypoalgesia, as well as tactile hypoesthesia in diabetic mice.

### In vivo effects of benztropine on sensory and neuromuscular excitability of oxaliplatin-treated and diabetic mice

**Fig. 4:**
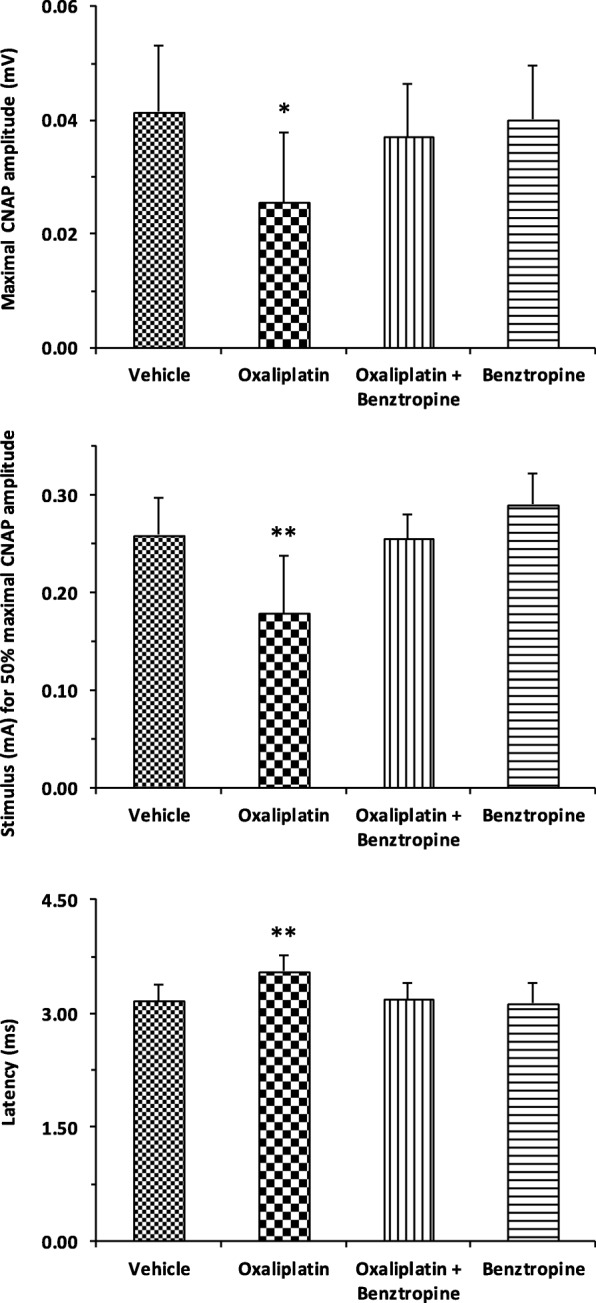
In vivo effects of oxaliplatin and benztropine on mouse sensory excitability variables. Histograms of mean values ± SD of maximal CNAP peak amplitude (upper panel), stimulus intensity necessary to evoke 50% of maximal CNAP amplitude (middle panel), and latency (lower panel), determined from recordings at the tail base in response to caudal nerve stimulation in mice treated for 6 weeks with vehicle (*n* = 9), oxaliplatin (*n* = 10), oxaliplatin plus benztropine (*n* = 10) or benztropine alone (*n* = 10). *p < 0.05, **p < 0.01 versus vehicle

Oxaliplatin-treated mice presented significant alterations, consistent with membrane hyperexcitability, in sensory excitability variables, compared with control animals (i.e.*,* mice injected with vehicle). In particular, the maximal CNAP amplitude was significantly reduced in oxaliplatin-treated mice compared to control mice (0.026 ± 0.012 with oxaliplatin versus 0.041 ± 0.012 with vehicle, *p* = 0.0137). Associating benztropine to the chemotherapy abrogated this reduction (0.037 ± 0.009 with oxaliplatin plus benztropine versus 0.041 ± 0.012 with vehicle, *p* = 0.4060), while benztropine alone did not alter maximal CNAP (0.040 ± 0.009 with benztropine versus 0.041 ± 0.012 with vehicle, *p* = 0.8096) (Fig. 4, upper panel). The stimulus intensity required to give 50% of maximal CNAP amplitude was also altered in mice treated with oxaliplatin (0.179 ± 0.059 with oxaliplatin versus 0.259 ± 0.038 with vehicle, *p* = 0.0041). Associating benztropine to the chemotherapy abrogated this reduction (0.256 ± 0.024 with oxaliplatin plus benztropine versus 0.259 ± 0.038 with vehicle, *p* = 0.8090), while benztropine alone did not alter this variable (0.290 ± 0.033 with benztropine versus 0.259 ± 0.038 with vehicle, *p* = 0.0909) (Fig. 4, middle panel). Finally, an increased latency was observed in oxaliplatin-treated mice (3.552 ± 0.202 with oxaliplatin versus 3.163 ± 0.218 with vehicle, *p* = 0.0014). This increase was not observed when mice treated with the chemotherapy also received benztropine (3.187 ± 0.220 with oxaliplatin plus benztropine versus 3.163 ± 0.218 with vehicle, *p* = 0.8284) or when mice received benztropine alone (3.137 ± 0.261 with benztropine versus 3.163 ± 0.218 with vehicle, *p* = 0.8230) (Fig. 4, lower panel). It is worth noting that the sensory alterations detected in oxaliplatin-treated mice were consistent with a decreased nerve conduction velocity, suggesting an apparent reduction in the number of fast-conducting fibers or decrease of density and/or functioning of transient sodium channels, and a modification in the voltage dependence of these channels.

Oxaliplatin-treated and diabetic mice also presented significant alterations, consistent with membrane hyperexcitability, in neuromuscular (motor) excitability waveforms and derived variables, compared with animals injected with vehicle (Additional file 1 and 2: Figure S1 and S2; Additional file 3: Table S1). These alterations mainly consisted of *(i)* an enhanced CMAP amplitude and a reduced stimulus intensity to evoke 50% of maximal CMAP amplitude, suggesting an apparent decreased density and/or functioning of fast potassium channels and modification in the voltage dependence of transient sodium channels, respectively, with no change in the latency, i.e.*,* no modification in the neurotransmission velocity; *(ii)* reduced minimum and hyperpolarizing slopes of the current–threshold relationship, indicating decreased density and/or functioning of cyclic nucleotide-gated channels; *(iii)* increased threshold changes in response to depolarizing and/or hyperpolarizing currents (threshold electrotonus), likely caused by reduced density and/or functioning of potassium channels; and *(iv)* lower superexcitability (recovery cycle), reflecting again fast potassium channel dysfunction. These alterations were not detected, or were greatly reduced, in oxaliplatin-treated and diabetic mice injected with benztropine, or in animals administered with benztropine alone.

### In vitro effects of oxaliplatin and benztropine on excitability of mouse DRG sensory neurons

**Fig. 5:**
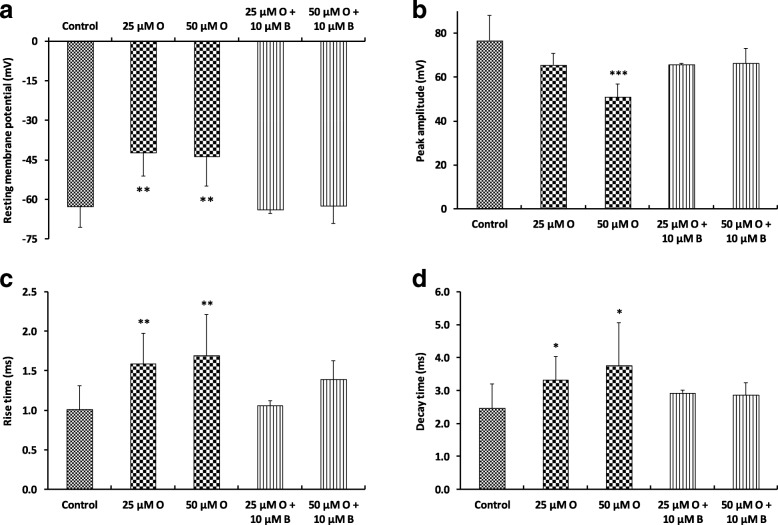
In vitro effects of oxaliplatin, associated or not with benztropine, on excitability of mouse DRG sensory neurons, using whole-cell patch-clamp technique. Resting membrane potential (**a**), and action potential peak amplitude (**b**), rise time (**c**) and decay time (**d**) measured from recordings performed on DRG neurons before (control) and 10–20 min after addition of first 25–50 μM oxaliplatin (O) and then 25–50 μM oxaliplatin plus 10 μM benztropine (B) to the standard physiological medium. Mean ± SD of 5–18 neurons. *p = 0.010–0.014, **p = 0.002–0.005 and ***p < 0.001 versus control

The in vitro effects of oxaliplatin, associated or not with benztropine, were assessed on the resting membrane and action potentials recorded from primary cultures of mouse DRG sensory neurons, using whole-cell patch-clamp technique (Fig. 5). Recordings were performed on relatively small neurons since their mean ± SD cell diameter (determined from their membrane capacity) was 21.6 ± 4.5 μm (*n* = 18).

Under control conditions, two types of responses were recorded from neurons stimulated by 100-ms current test-pulses: a single action potential (tonic response) from 39% (7/18) of cells, and more than one action potential (phasic response) from 61% (11/18) of cells. The mean ± SD ratio of the number of action potentials on the current intensity necessary to evoke action potentials was 0.007 ± 0.001 pA^− 1^ (*n* = 18). This ratio was significantly increased (*p* = 0.024) to 0.011 ± 0.002 pA^− 1^ (*n* = 11) in the presence of 50 μM oxaliplatin, due to both an increased number of action potentials in response to 100-ms current test-pulses and a decreased current intensity necessary to evoke action potentials, indicating membrane hyperexcitability of these neurons. When oxaliplatin (50 μM) was added together with benztropine (10 μM) to the standard external medium, the ratio returned to 0.005 ± 0.002 pA^− 1^ (*n* = 9), i.e.*,* mean ± SD values were not statistically different (*p* = 0.148) from those determined under control conditions.

The addition of oxaliplatin (25 or 50 μM) to the external medium bathing the neurons produced *(i)* about 20-mV membrane depolarization identified by a reduced resting membrane potential, *(ii)* a significant decreased peak amplitude, and *(iii)* significant increased rise and decay times of action potentials, compared to control conditions. These modifications, indicating alterations in the density and/or functioning of both sodium and potassium channels, were greatly reduced, if not completely reversed, when oxaliplatin was added together with benztropine (10 μM) to the external standard medium.

### Ex vivo effects of oxaliplatin and benztropine on mouse sciatic nerve fiber morphology and myelin protein content

**Fig. 6:**
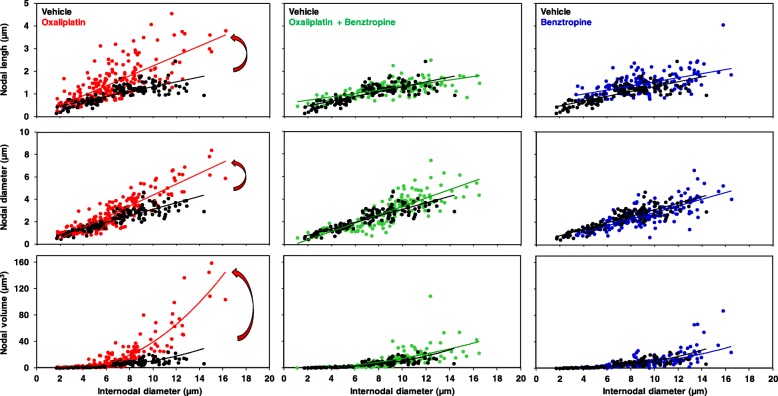
Ex vivo effects of oxaliplatin and benztropine on the morphology of myelinated axons isolated from mouse sciatic nerves, using confocal microscopy. Representations of nodal length, diameter and volume, as functions of internodal diameter, of myelinated axons isolated from mice (*n* = 4 in each group) injected with vehicle (black closed circles, *n* = 137), oxaliplatin (red closed circles, *n* = 206), oxaliplatin plus benztropine (green closed circles, *n* = 150) or benztropine alone (blue closed circles, *n* = 160) for 6 weeks. The curves represent the linear (upper and middle panels) or non-linear (lower panels) fits of data points with R^2^ (correlation coefficients) between 0.441 and 0.877. In left panels, the arrows underline the effects of oxaliplatin

The morphology of myelinated axons of mouse sciatic nerves was assessed using confocal microscopy. Quantification of morphometric parameters of single myelinated axons revealed a significant increase in the nodal diameter, length and volume, as functions of the internodal diameter, in mice injected for 6 weeks with oxaliplatin, compared to vehicle-treated animals (Fig. 6, left panels; Additional file 4: Table S2). These results are likely the consequence of oxaliplatin-induced membrane hyperexcitability. In these animals, a reduction of the internodal diameter was also observed, which may reflect either a preferential loss of large myelinated nerve fibers or an alteration in myelin sheath layers surrounding the axons. These alterations of morphometric parameters were greatly reduced, if not absent, in mice injected for 6 weeks with oxaliplatin plus benztropine (Fig. 6, middle panels; Additional file 4: Table S2) or benztropine alone (Fig. 6, right panels; Additional file 4: Table S2).

**Fig. 7:**
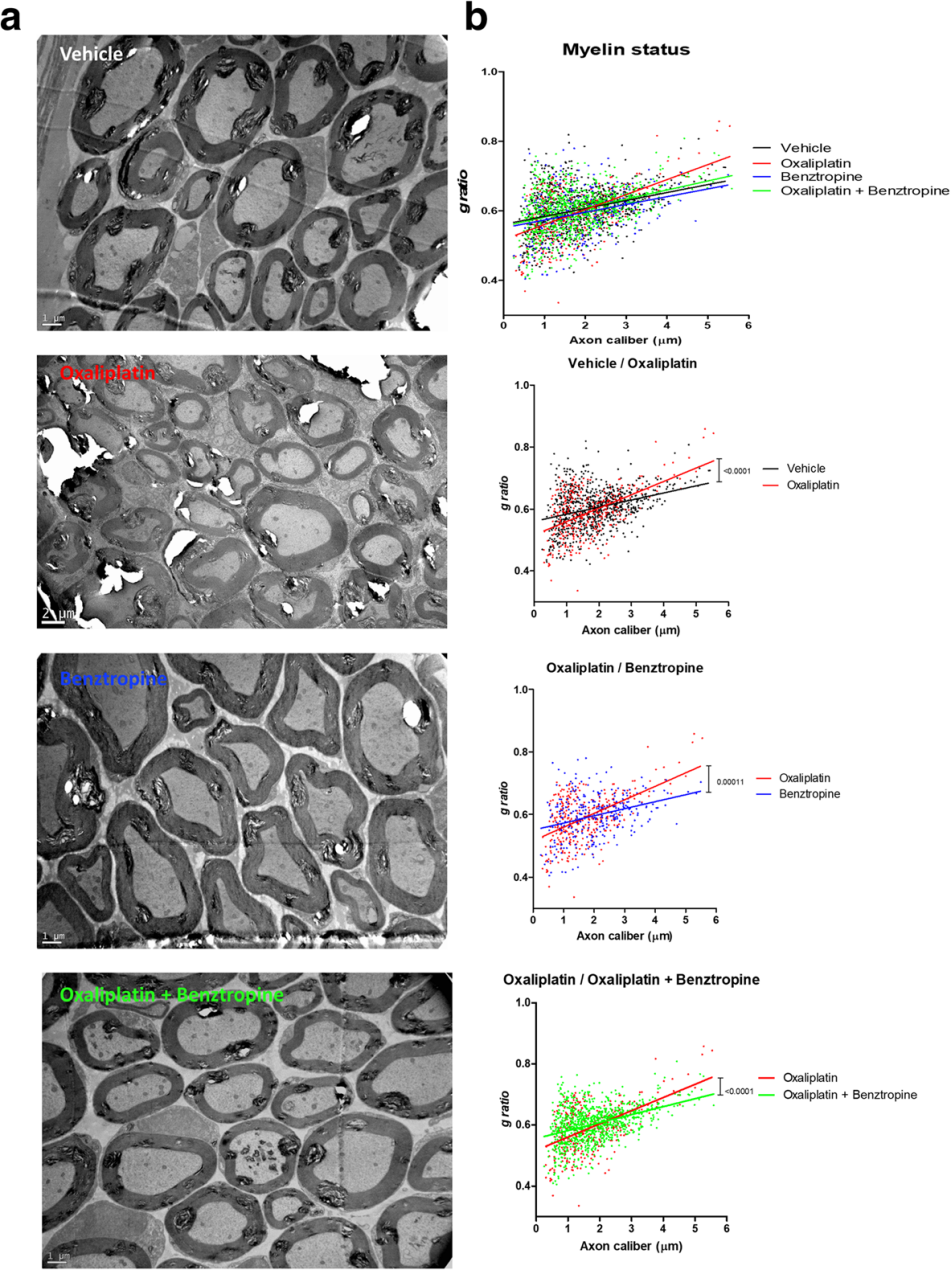
Effect of benztropine on demyelination and axonal atrophy in oxaliplatin-treated mice. **a** Representative images of EM micrographs of ultrathin cross-sections of sciatic nerves from vehicle, oxaliplatin, benztropine and oxaliplatin plus benztropine animals at 6 weeks. **b** Quantification of myelin state though g-ratio analysis reported to axonal caliber. At least 200 axons per animal (*n* = 2–3) were analyzed. The curves are linear regression fits of data points. *p < 0.05, **p < 0.01

The myelin sheaths of sciatic nerves from experimental and control groups of mice were also assessed using electronic microscopy. Analyses revealed a demyelination in oxaliplatin-treated mice compared to animals injected with vehicle, oxaliplatin plus benztropine or benztropine alone (Fig. 7). Semi-automated computerized measurement of myelin thickness allowed the quantification of the profound demyelination in sciatic nerves from oxaliplatin mice (0.0344 ± 0.0037 with oxaliplatin versus 0.0233 ± 0.0023 with vehicle, *p* = 0.007). Associating benztropine to the chemotherapy abrogated the reduction in myelin sheath thickness observed in oxaliplatin mice (0.0261 ± 0.0019 with oxaliplatin plus benztropine versus 0.0233 ± 0.0023 with vehicle, *p* = 0.346). Benztropine did not impair myelin formation nor did it lead to excessive myelination (0.0224 ± 0.0038 with benztropine versus 0.0233 ± 0.0023 with vehicle, *p* = 0.842).

### Ex vivo effects of oxaliplatin and benztropine on mouse cutaneous nerve fiber density

**Fig. 8:**
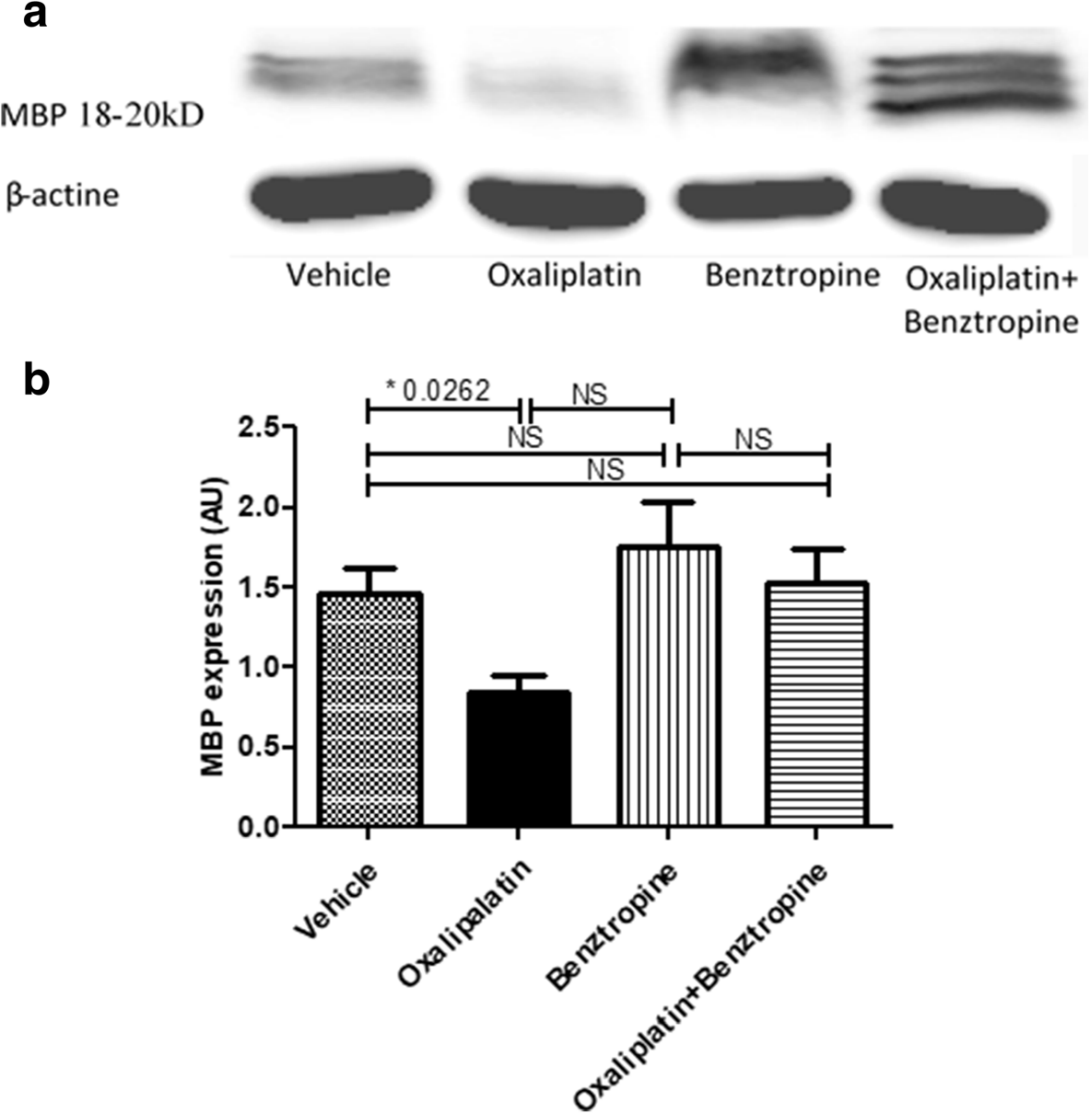
Effect of benztropine cutaneous nerve fiber density reduction induced by oxaliplatin. **a** Representative images of staining with PGP9.5 of cutaneous nerve fibers. **b** Analysis of cutaneous nerve fiber density from paw skin samples (6 μm) of mice treated for 6 weeks with vehicle, oxaliplatin, oxaliplatin plus benztropine or benztropine alone. Mean ± SEM of 8 mice. *p < 0.05, **p < 0.01. NS: non-significant. Scale bar = 50 μm

The density of cutaneous nerve fibers was examined in the paws of mice injected with vehicle, oxaliplatin, oxaliplatin plus benztropine or benztropine alone for 6–8 weeks (Fig. 8). Staining of the nerve fibers with PGP9.5 antibody revealed a reduced cutaneous nerve fiber density in paw skin of oxaliplatin-treated mice (7.67 ± 0.99 with oxaliplatin versus 16.33 ± 1.87 with vehicle, *p* = 0.0022). Benztropine abrogated this alteration in oxaliplatin-treated mice since the density of PGP9.5-stained nerves was maintained in these animals (13.50 ± 1.56 with oxaliplatin plus benztropine versus 16.33 ± 1.87 with vehicle, *p* = 0.2728). Benztropine alone did not alter cutaneous nerve fiber’s integrity (15.00 ± 2.85 with benztropine versus 16.33 ± 1.87 with vehicle, *p* = 0.7042).

### Ex vivo study of myelin protein content in sciatic nerves

**Fig. 9:**
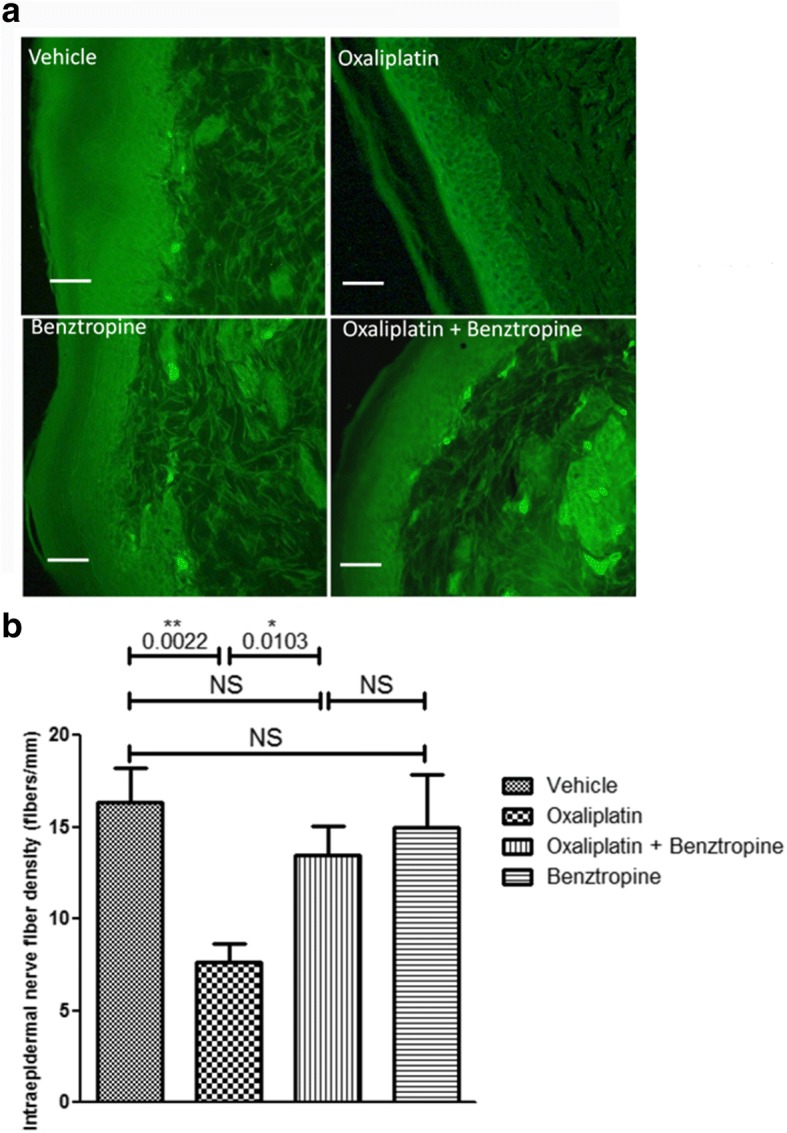
Effect of benztropine on MBP expression in the sciatic nerves of oxaliplatin-treated mice. Western blot analyses of total protein lysates from brain sciatic nerves of control, and treated mice. **a** panel shows detection of MBP and **b** panel shows anti-β-actin for loading control. NS: non-significant

The myelin basic protein (MBP) expression was reduced in oxaliplatin-treated mice (0.5825 ± 0.1014 with oxaliplatin versus 0.7125 ± 0.1409 with vehicle, *p* = 0.4823), while co-administration of benztropine with the chemotherapy rescued this reduced expression of this key myelin protein (0.6767 ± 0.1172 with oxaliplatin plus benztropine versus 0.7125 ± 0.1409 with vehicle, *p* = 0.8603). It is worth nothing that MBP expression was identical in mice receiving benztropine alone and control animals (0.7075 ± 0.2781 with benztropine versus 0.7125 ± 0.1409 with vehicle, *p* = 0.8603) (Fig. 9).

### Ex vivo effects of oxaliplatin and benztropine on systemic inflammatory markers

**Fig. 10:**
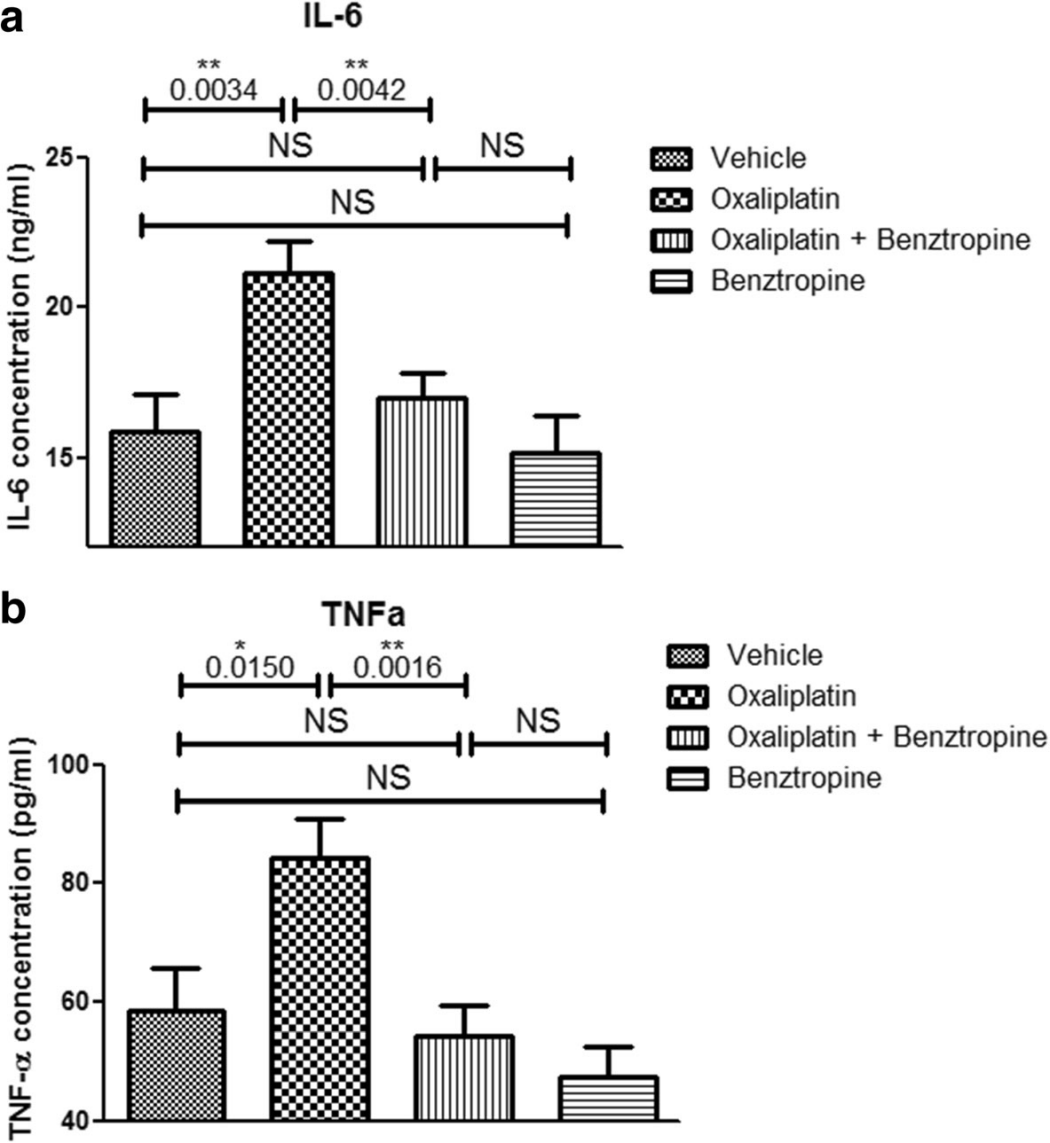
Sera inflammatory markers. **a** ELISA-quantified levels of IL-6 in sera from mice treated for 6 weeks with vehicle, oxaliplatin, oxaliplatin plus benztropine or benztropine alone. **b** ELISA-quantified levels of TNF-α in sera from mice treated for 6 weeks with vehicle, oxaliplatin, oxaliplatin plus /benztropine or benztropine alone. Data are mean ± SEM of 8 mice. *p < 0.05, **p < 0.01, ***p < 0.001 versus vehicle. NS: non-significant

In order to evaluate the systemic effects of benztropine on pro-inflammatory cytokine levels, serum samples were analyzed (Fig. 10). Sera IL-6 levels were significantly increased in the oxaliplatin-treated group (21.14 ± 1.07 ng/mL with oxaliplatin versus 15.87 ± 1.27 ng/mL with vehicle, *p* = 0.0034). Associating benztropine to the chemotherapy prevented this systemic inflammation (16.97 ± 0.81 ng/mL with oxaliplatin plus benztropine versus 15.87 ± 1.27 ng/mL with vehicle, *p* = 0.4674, and versus 21.14 ± 1.07 ng/mL with oxaliplatin, *p* = 0.0042). Benztropine alone did not induce any changes in IL-6 levels (15.12 ± 1.28 ng/mL with benztropine versus 15.87 ± 1.27 ng/mL with vehicle, *p* = 0.6829). Sera TNF-α levels were also significantly increased in the oxaliplatin-treated group compared to the control group (84.18 ± 6.86 pg/mL with oxaliplatin versus 58.36 ± 7.27 pg/mL with vehicle, *p* = 0.0150). Similar to its effects on IL-6 levels, benztropine also prevented the significant increase in TNF-α levels observed in oxaliplatin-treated-animals (54.36 ± 5.17 pg/mL with oxaliplatin plus benztropine versus 58.36 ± 7.27 pg/mL with vehicle, *p* = 0.6570, and versus 84.18 ± 6.86 pg/mL with oxaliplatin, *p* = 0.0016). Benztropine on its own induced a slight, but non-significant, decrease in TNF-α levels (47.30 ± 5.17 pg/mL with benztropine versus 58.36 ± 7.27 pg/mL with vehicle, *p* = 0.2246).

### In vivo effects of benztropine on oxaliplatin-induced tumor growth in mice

**Fig. 11:**
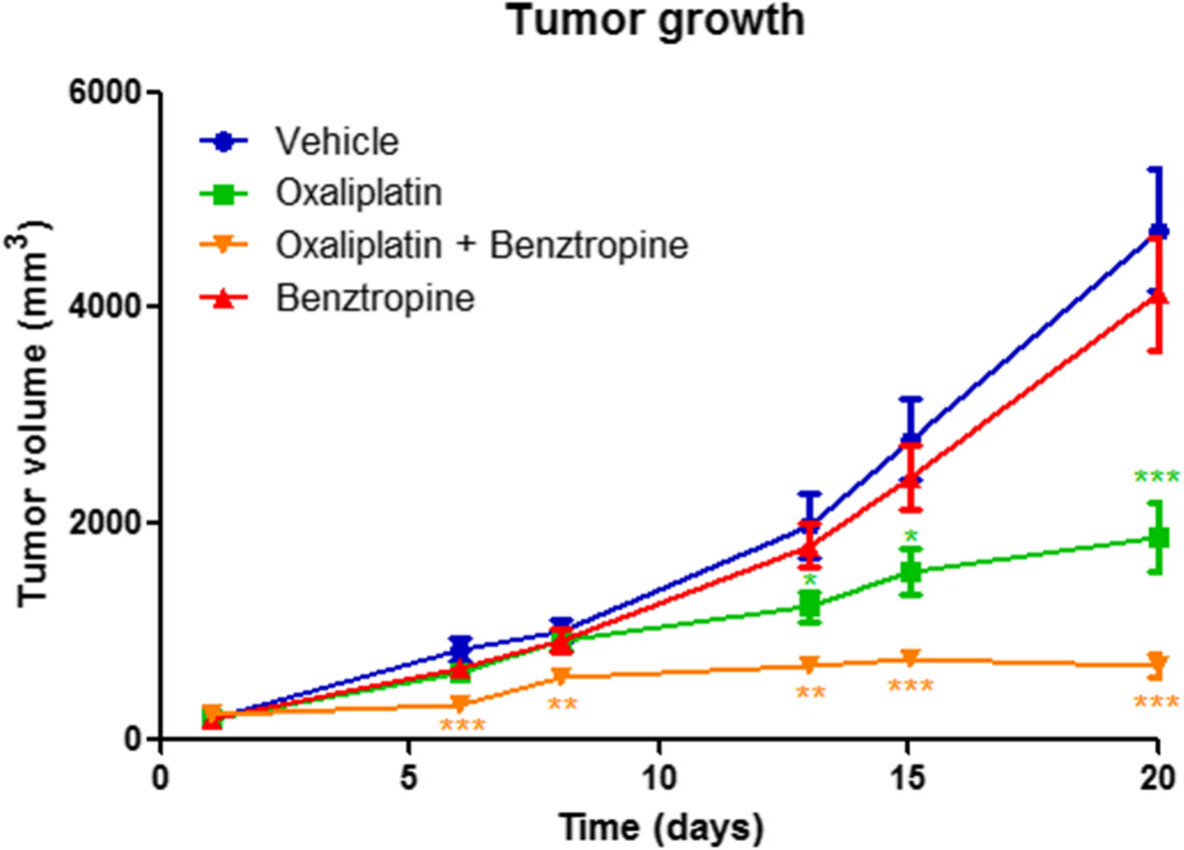
Benztropine prevents tumor growth and displays a synergistic antitumoral effect when associated with oxaliplatin in an ectopic model of colorectal cancer. Tumor size in mice injected subcutaneously into the back with 106 CT26 cells and treated with vehicle, oxaliplatin, oxaliplatin associated with benztropine or benztropine alone. Data are mean ± SEM of 8 tumor volume under each condition. *p < 0.05, **p < 0.01, ***p < 0.001 versus vehicle

Results obtained from neurological tests provided a rationale to use benztropine as a neuroprotective drug. However, further data regarding its effect on oxaliplatin efficacy had to be gathered to guarantee its safety in addressing the primary aspect of the condition which is tumor growth (Fig. 11). From day 6 of treatment, benztropine displayed antitumor efficacy upon association with oxaliplatin (tumor size of 333.5 ± 42.3 mm^3^ with oxaliplatin plus benztropine versus 832.1 ± 105.8 mm^3^ with vehicle, *p* = 0.0009). Moreover, from day 6 treatment, benztropine potentiated the chemotherapy response (tumor size of 333.5 ± 42.3 mm^3^ with oxaliplatin plus benztropine versus 624.8 ± 69.8 mm^3^ with oxaliplatin, *p* = 0.0039), while oxaliplatin alone had not yet demonstrated antitumor efficacy (tumor size of 624.8 ± 69.8 mm^3^ with oxaliplatin versus 832.1 ± 105.8 mm^3^ with vehicle, *p* = 0.1279). At the end of the experiment, when the largest tumors had reached ethical guidelines’ endpoint, mice which received benztropine associated with oxaliplatin presented the lowest tumor burden (687.5 ± 103.3 mm^3^ with oxaliplatin plus benztropine versus 4721.0 ± 560.0 mm^3^ with vehicle, *p* < 0.0001). In addition, mice which received benztropine alone displayed smaller, although non-significant, tumors compared to control mice (4132.0 ± 522.8 mm^3^ with benztropine versus 4721.0 ± 560.0 mm^3^ with vehicle, *p* = 0.4571).

### In vitro effects of oxaliplatin associated or not with benztropine on cultured cell viability

**Fig. 12:**
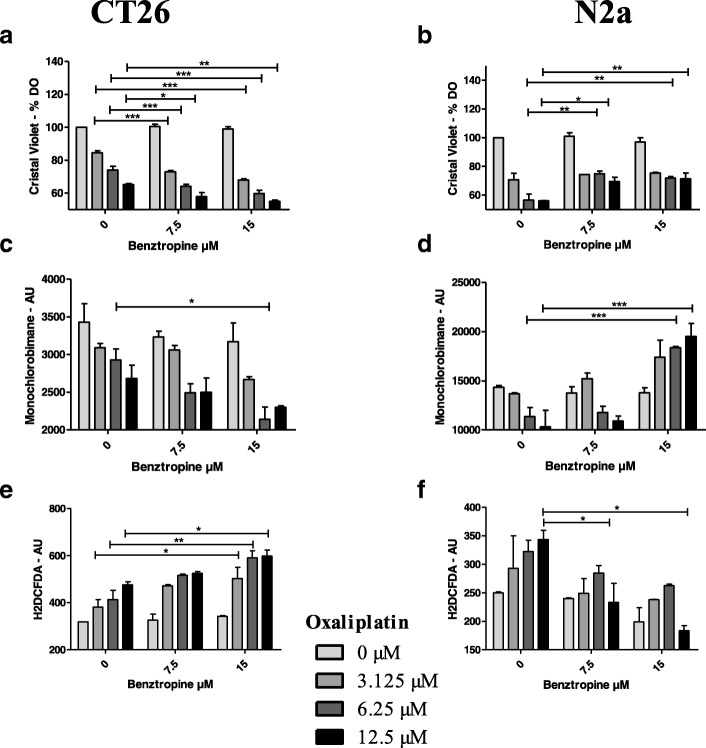
In vitro effects of oxaliplatin associated or not with benztropine on cell viability, GSH and ROS production. Viability was expressed as percent ± SEM versus cells in culture medium alone (100% viability) in CT26 cells (**a**) and N2a cells (**b**). GSH was measured using the monochlorobimane dye in CT26 cells (**c**) and N2a cells (**d**). ROS generation was measured using H2DCFDA fluorescent emission when converted to the highly fluorescent DCF in CT26 cells (**e**) and N2a cells (**f**). Data from at least 4 independent experiments have been pooled and were expressed as means ± SEM of triplicates. *p < 0.05, **p < 0.01 versus oxaliplatin

The in vitro neuroprotective potential of benztropine on oxaliplatin-induced peripheral neuropathies was assessed on N2a neuron-like cells. The effects of benztropine on oxaliplatin-induced anti-tumor effect was also evaluated in vitro on CT26 colon carcinoma cell lines. Our results indicate that oxaliplatin has a cytotoxic effect on both N2a and CT26 cells. Benztropine modulates cell viability in a cell-type-dependent manner. Benztropine potentiated oxaliplatin cytotoxicity on CT26 cells (Fig. 12a). Inversely, in benztropine-treated neuron-like N2a cells, the cytotoxicity of oxaliplatin was reduced with a viability increased from 56% without benztropine to 70% with benztropine (Fig. 12b). Moreover, in cells treated with 6.25 μM oxaliplatin, co-incubation with 15 μM of benztropine induced a significant decrease of GSH in CT26 cells (2141 ± 120 versus 2931 ± 142 in untreated cells, *p* < 0.05, Fig. 12c) but a significant increase of GSH in N2a (18,344 ± 634 versus 11,355 ± 934 in untreated cells, *p* < 0.001, Fig. 12d). ROS production evidenced by H2DCFDA staining was stimulated in both cell types treated with oxaliplatin. However, benztropine at a concentration of 7.5 and 15 μM decreased oxaliplatin-induced ROS production in N2a cells (*p* < 0.05) (Fig. 12f) and, in contrast, 15 μM of benztropine increased oxaliplatin-induced ROS production in CT26 cells (p < 0.05 to *p* < 0.01) (Fig. 12e).

## Discussion

Peripheral neuropathy is one of the most prevalent neurologic conditions encountered by physicians of all specialties [88] and is frequently associated with viral, toxic or metabolic etiologies [60]. Despite its high prevalence, no efficient curative treatment is available to decrease peripheral neuropathic pain and disability [24]. In this report, we demonstrate that benztropine, an inhibitor of acetylcholine (ACh) muscarinic M1 and M3 receptors (mAChR) [22], improves both acute and chronic clinical symptoms of experimental peripheral neuropathies in mice induced either by treatment with the chemotherapeutic drug oxaliplatin or with streptozotocin (STZ) in a model of diabetes-induced peripheral neuropathy [81, 86].

In order to better understand the mechanisms of this protection at neuronal levels, we performed electrophysiological tests to evaluate sensory and neuromuscular excitability. Oxaliplatin-treated mice presented significant reductions of the maximal CNAP amplitude and of the stimulus intensity required to give 50% of maximal CNAP as well as an increase in latency, both these signs being associated with membrane hyperexcitability. The sensory alterations detected in oxaliplatin-treated mice were consistent with a decreased nerve conduction velocity, suggesting an apparent reduction in the number of fast-conducting fibers or a decrease of density and/or functioning of transient sodium channels, and a modification in the voltage dependence of these channels. These alterations were all prevented by treatment with benztropine. The in vitro effects of oxaliplatin on the resting membrane and action potentials recorded from primary cultures of mouse DRG sensory neurons using whole-cell patch-clamp showed modifications characteristic of alterations in the density and/or functioning of both sodium and potassium channels. These alterations were greatly reduced, if not completely reversed, when the anticancer agent was added together with benztropine (10 μM) to the external standard medium.

Oxaliplatin is known to exhibit a tetrodotoxin-like inhibitory effect on neuronal voltage-gated sodium (Na+) channels [1, 8, 89]. It remarkably slows their inactivation and reduces the peak Na + current, leading to an increase in the duration of the relative refractory period of sensory neurons that become hyperexcitable. Oxaliplatin may also affect the Na + channels indirectly via the chelation of extracellular calcium ions by its metabolite oxalate (diaminocyclohexane-platinum-C2O4) [1]. Peripheral nerve axonal excitability studies performed after oxaliplatin administration in vivo have revealed acute abnormalities in sensory nerve function related to Na + channel dysfunction, including decreased refractoriness and increased superexcitability [63]. The effects of oxaliplatin on the Nav1.6 voltage-gated Na + channel isoforms have been associated with the development of unique neuropathy symptoms such as cold-aggravated peripheral pain [23, 76]. In rat hippocampal neurons, muscarinic receptor agonists modulate Na + channel activity through activation of PKC [12]. In the periphery, the implication of PKC activation in nociceptive neurons has been largely studied and linked to hyperexcitability and hyperalgesia through upregulation of both Nav1.8 and Nav1.9 [47, 90]. Blocking PKC by muscarinic antagonists could be relevant to prevent peripheral neuropathies, as PKC inhibition has been shown to prevent hyperalgesia in an in vivo model of diabetic neuropathy [41].

Kagiava et al. [40] suggested that altered voltage-gated potassium channel activity may also be involved in oxaliplatin-induced neurotoxicity. Oxaliplatin was found to cause broadening of action potentials and repetitive firing, suggesting its antagonistic effect on fast and slow neuronal potassium channels. Many sensory neurons in rat dorsal root ganglia express Kv7.2, Kv7.3 and Kv7.5. These channels are known as M channels and are closed by receptors coupled to Gq such as M1 and M3 muscarinic receptors. This blockade increases neuronal excitability and underlies some forms of cholinergic excitation. By contrast, their activation attenuates sensory Aδ and C-fibre discharges induced by heat stimulation when applied to the *peripheral* endings of sensory fibres in the isolated rat skin nerve preparation [64], and increases the threshold for C-fiber stimulation in human sural nerves [48]. These experiments clearly demonstrate the presence of Kv7/M-channels at various sites along the sensory neuraxis and also indicate the therapeutic potential of enhancing their activity [10]. Interestingly, Sittl et al. [75] showed that enhancement of axonal potassium conductance by flupirtine, a Kv7/M-channel enhancer could reduce oxaliplatin-induced peripheral nerve hyperexcitability, supporting our findings that blockade of muscarinic receptor M1 and M3 by benztropine can produce the same effects.

Oxaliplatin-treated and diabetic mice also presented significant alterations, consistent with membrane hyperexcitability, in neuromuscular (motor) excitability waveforms and derived variables, compared with animals injected with vehicle, suggesting an apparent decreased density and/or functioning of fast potassium channels and modification in the voltage dependence of transient sodium channels, respectively; decreased density and/or functioning of cyclic nucleotide-gated channels; reduced density and/or functioning of potassium channels. These alterations were not detected, or were greatly reduced, in oxaliplatin-treated and diabetic mice injected with benztropine, or in animals administered with benztropine alone. At the neuromuscular levels, oxaliplatin has been shown to increase both evoked and spontaneous neurotransmitter release in the motor nerve terminal of a phrenic nerve hemidiaphragm preparation [89]. The hyperexcitability effects of oxaliplatin on the mammalian neuromuscular junction appear related to a mechanism which delays entry of Na + channels into an inactivated state and, to a lesser extent, to a reduced K+ channel activity, both of which can be prevented by benztropine.

Electronic microscopy of the myelin sheaths of the sciatic nerves revealed a demyelination in oxaliplatin-treated mice compared to animals injected with vehicle alone, oxaliplatin plus benztropine or benztropine alone. Confocal microscopy of mouse sciatic nerves confirms the alteration of the morphology of myelinated axons with a significant increase in the nodal diameter, length and volume, as functions of the internodal diameter, in oxaliplatin-treated mice compared to vehicle-treated animals. These results are likely the consequence of oxaliplatin-induced membrane hyperexcitability as previously described [8]. In these animals, a reduction of the internodal diameter was also observed, which may reflect either a preferential loss of large myelinated nerve fibers or an alteration in myelin sheath layers surrounding the axons. The drop in MBP expression in oxaliplatin-treated animals confirmed the severe reduction of neuron myelination in DRG and sciatic nerves. These alterations of morphometric parameters were greatly reduced, if not absent, in mice co-injected with oxaliplatin and benztropine. In a recent study, Imai and coworkers [37] showed that treatment with oxaliplatin induced cytotoxicity of myelin-forming Schwann cells accompanied by mitochondrial dysfunction at concentrations lower than those impairing DRG neurons. These direct effects of oxaliplatin on Schwann cells might be an underlying cause of CIPN in addition to its direct toxicity in peripheral neurons. Schwann cells express muscarinic receptors M1, M2, M3, and M4 [27, 55] and localize to the axon-Schwann cell boundary [67]. Activation of AChR on Schwann cells modified the myelin sheet by altering the viscosity of the myelin membrane [84] that can be prevented by muscarinic receptor antagonists.

A severe reduction in the number of cutaneous nerve fibers is frequently observed in humans and mice treated with oxaliplatin as a result of neuronal toxicity of the platinum compound. Interestingly, benztropine also prevented the drop in neuronal density in the paws of mice injected with oxaliplatin. It has recently been demonstrated that neurite outgrowth is controlled by muscarinic receptors through regulation of mitochondrial function. M1R-deficient mice rended diabetic with STZ are protected from physiological and structural indices of sensory neuropathy and pharmacological blockade of M1R using antimuscarinic drugs prevented or reversed indices of diabetic or chemotherapy-induced peripheral neuropathy [11].

Proinflammatory cytokines like TNFα or IL-6 contribute to axonal damage, alteration of the myelin structure and voltage dependent channel integrity but also modulate spontaneous nociceptor sensitivity and activity by increasing Na + and Ca2+ currents at the nociceptor peripheral terminals, which results in an increased membrane excitability, and a reduction in pain threshold and peripheral sensitization [43]^,^ [20, 71]. The pain sensation in distal extremities has been attributed to dysfunction of small myelinated Aδ or unmyelinated C-fibers [28, 85]. Sensitizing uninjured adjacent nerve fibers (nociceptors) or sensory neurons by proinflammatory cytokines plays a critical role in the development of the chemotherapy- and diabetic-induced painful peripheral neuropathy as evidenced by numerous clinical and experimental studies [57, 71]. Indeed, following intravenous administration of chemotherapy drugs, an important activation of Schwann cells along with a massive infiltration of activated macrophages in the DRG and in peripheral nerves leads to a subsequent production and secretion of inflammatory cytokines like TNFα, IL-1β and IL-6; promoting neuroinflammation with allodynia and hyperalgesia [65]. Injection of an anti-IL-6 neutralizing antibody alleviated pain-related behaviors [59, 83] and a recent clinical study reported that IL-6 levels were significantly higher after the conclusion of chemotherapy in breast cancer patients with CIPN than in those without CIPN, providing the first clinical evidence of the involvement of IL-6 in CIPN [78]. In diabetes, an enhanced expression of the NF-κB-derived cytokine TNFα in the sciatic nerve of diabetic rats and mice was associated with decreased expression of myelin basic protein and with both large and small nerve fiber dysfunction, as documented by reductions in the motor and sensory nerve conduction velocities and in the intraepidermal nerve fiber density in the diabetic animals [42]. These neuronal dysfunctions were all reverted by blocking TNF with a recombinant human TNF receptor–antibody fusion protein [73]. Interestingly, neurons and immune cells, especially macrophages, express all five muscarinic receptors and their stimulation promotes a pro-inflammatory reaction while blocking the M1 and M3 receptors has been shown to reduce the synthesis of the pro-inflammatory cytokines IL-6 and TNFα [29].

In addressing the primary aspect of the condition, which is tumor growth, we found that mice receiving benztropine alone had a reduced size of tumors compared to untreated animals and when associated with oxaliplatin presented the lowest tumor burden compared to mice treated with oxaliplatin alone. These data are in line with the role of muscarinic receptors on tumor growth and especially on colon cancer progression. Indeed, the levels of expression and activation of M3 muscarinic receptors in colon cancer cells are very high and associated with increased tumor cell proliferation by activation of the MAPKinase pathways and invasiveness by increasing MMP1 release. All of these effects are reduced by M3R antagonists [26].

Benztropine exerts an original effect on neuronal cells by increasing the level of reduced glutathione thus reducing ROS levels induced by oxaliplatin and cell death. Blocking muscarinic receptors has already been associated with a reduction in ROS release, prevention of glutathione depletion and enhanced viability in various normal non tumoral cell types [82]. By contrast, benztropine potentiates the cytotoxic effect of oxaliplatin on tumor cells via a drop in GSH levels and an increase in H_2_O_2_ production, in line with the capacity of benztropine to reduce neuronal toxicity while maintaining the anti-tumor efficacy of oxaliplatin. Activation of the M1 muscarinic receptor by various agonists decreases SOD activities and induces superoxide anion production in neuronal cells [56]. Interestingly, superoxide anions have been shown to be particularly cytotoxic for neurons upon exposure to oxaliplatin, as superoxide dismutase mimics prevent oxaliplatin-induced neuropathies while increasing the anti-tumoral effect of the chemotherapy on colon cancer cells [18]. SOD increase by M1 blockade leads to superoxide anions dismutation into H_2_O_2_, whose cytotoxicity towards tumor cells adds up to that of oxaliplatin. The differences observed between normal and tumor cells fate upon oxaliplatin and benztropine exposure can be related to their different basal levels of GSH and responses towards a H_2_O_2_-mediated stress [3, 49, 62, 80]. Tumor cells are more sensitive to ROS-induced cell death than normal cells due to increased metabolism of tumor cells and antioxidant defense exhaustion. Indeed, in normal cells, similar levels of H_2_O_2_ that kill tumor cells favor cellular viability and proliferation through adaptation and mobilization of antioxidant defenses, usually through NRF2 induction [33]. Furthermore, GSH homeostasis is of prime importance for neurons as their depletion in this antioxidant molecule ultimately leads to their senescence mediated by oxidative stress [7]. Several studies concur with the observation that GSH-mediated detoxification of H_2_O_2_ is paramount to address neurologic disorders, may they be central or peripheral [5, 69]. In clinical settings, GSH infusions significantly reduce the severity of the neurodegenerative side effects of oxaliplatin [14].

## Conclusion

In the present study, we show that benztropine inhibits the oxaliplatin adverse effects mainly focusing on its anti-muscarinic action. However, benztropine also presents additional pharmacologic properties. Indeed, benztropine is a H1 receptor antagonist and an inhibitor of the dopamine re-uptake. Those properties are relevant since Khalilzadeh E et al. demonstrated that H1 receptor antagonists could attenuate the mechanical allodynia and prevented cold plate avoidance in a rat model of neuropathic pain [44]. Such observation has never been made in CIPN. However, we cannot rule out that the improvement of neuropathy we observed in our model was due to, at least in part, the anti-histaminic effect of benztropine. Moreover, benztropine also acts as a dopamine re-uptake inhibitor. It has been shown by Hache G et al. that administration of drugs that enhance the activity of dopamine neurotransmission also provide antinociceptive effects [35]. Therefore, we cannot exclude that the dopamine re-uptake inhibition by benztropine may participate in the prevention of oxaliplatin-induced peripheral neuropathy.

Finally, even though M1 and M3 muscarinic receptors inhibition remains the major mechanism regulated by benztropine to counteract CIPN and tumor growth, we cannot rule out that its effects on H1 histaminic receptors and dopamine re-uptake may also participate in the beneficial effects of benztropine. Therefore more detailed studies are needed to investigate the relative contribution of these pathways.

A limitation of this study is that results have been gained in animals only. However, the literature indicates that murine models of peripheral neuropathies are robust models for modeling human neuropathy [50, 76]. Indeed, the components of this murine neurodegeneration are also observed in humans and the neuroprotection conferred by molecules on these models is also observed in humans [18, 68]. This observation leads us to believe that the neuroprotection conferred by benztropine, which is currently indicated in Parkinson’s disease in the United States, could be transposable to patients suffering from peripheral neuropathies. These very encouraging results for benztropine deserve to lead to a study in humans in order to be able to address a problematic with clinical relevance. The results of this clinical trial could lead to the exploration of benztropine as a potentially neuroprotective agent in other pathologies of the PNS.

## Additional files

Additional file 1: **Figure S1.** Benztropine prevents the in vivo effects induced by oxaliplatin on mouse neuromuscular excitability curves. Excitability curves (means ± SD) were recorded at the plantar muscle in response to sciatic motor nerve stimulation of mice treated for 6 weeks with vehicle (black circles, *n* = 18), oxaliplatin (white circles, *n* = 14, left panels), oxaliplatin plus benztropine (white circles, *n* = 10, middle panels) or benztropine alone (white circles, *n* = 13, right panels). (C1) Current-threshold relationship [excitability modifications in response to depolarizing (up) and hyperpolarizing (down) currents], (C2) strength-duration relationship, (C3) threshold electrotonus in response to constant depolarizing (up) and hyperpolarizing (down) long-duration currents applied at sub-threshold intensity (± 40%), and (C4) recovery cycle. Note the absence of effect of oxaliplatin plus benztropine and benztropine alone, versus vehicle, on excitability waveforms. (TIF 1435 kb)

Additional file 2: **Figure S2.** Benztropine prevents the in vivo effects induced by diabetes on mouse neuromuscular excitability curves. Excitability curves (means ± SD) were recorded at the plantar muscle in response to sciatic motor nerve stimulation of mice treated for 6 weeks with non-diabetic vehicle (black circles, *n* = 18), diabetic vehicle (white circles, *n* = 16, left panels), and diabetic benztropine (white circles, *n* = 14, right panels). (C1) Current-threshold relationship [excitability modifications in response to depolarizing (up) and hyperpolarizing (down) currents], (C2) strength-duration relationship, (C3) threshold electrotonus in response to constant depolarizing (up) and hyperpolarizing (down) long-duration currents applied at sub-threshold intensity (± 40%), and (C4) recovery cycle. Note the absence of effect of diabetic benztropine, versus non-diabetic vehicle, on excitability waveforms. (TIF 1006 kb)

Additional file 3: : **Table S1.** Benztropine prevents the in vivo effects induced by oxaliplatin and diabetes on mouse neuromuscular excitability variables. Variables (means ± SD) derived from excitability curves established from plantar muscle recordings in response to sciatic motor nerve stimulation of mice treated with vehicle (*n* = 18), oxaliplatin (*n* = 14), oxaliplatin plus benztropine (*n* = 10), diabetic vehicle (*n* = 16), diabetic benztropine (*n* = 14) or benztropine alone (*n* = 13) for 6 weeks. (C0) Stimulus-response relationship, (C1) current-threshold relationship, (C2) strength-duration relationship, (C3) threshold electrotonus in response to constant depolarizing (TEd) and hyperpolarizing (TEh) long-duration currents applied at sub-threshold intensity (± 40%), and (C4) recovery cycle. *p < 0.05, **p < 0.01, ***p < 0.001, and ****p < 0.0001 versus vehicle (highlighted in grey). Note that, compared to vehicle; most if not all variables modified with oxaliplatin and diabetic vehicle remain unchanged with oxaliplatin plus benztropine, diabetic benztropine or benztropine alone. (TIF 593 kb)

Additional file 4: : **Table S2.** Comparison of morphometric parameters. Mean values ± SD of morphometric parameters of single myelinated axons isolated from sciatic nerves of mice injected with vehicle, oxaliplatin, oxaliplatin plus benztropine or benztropine alone, for 6 weeks (137–206 axons from 4 different mice under each condition). *P = 0.011–0.049, **P = 0.006 and ***P < 0.0001 versus vehicle (highlighted in grey). (TIF 155 kb)
