# Supplementary figures and images for: Preventive action of benztropine on platinum-induced peripheral neuropathies and tumor growth
Source: Acta Neuropathol Commun. 2019 Jan 18;7:9. doi: 10.1186/s40478-019-0657-y (PMC6337872; doi:10.1186/s40478-019-0657-y)

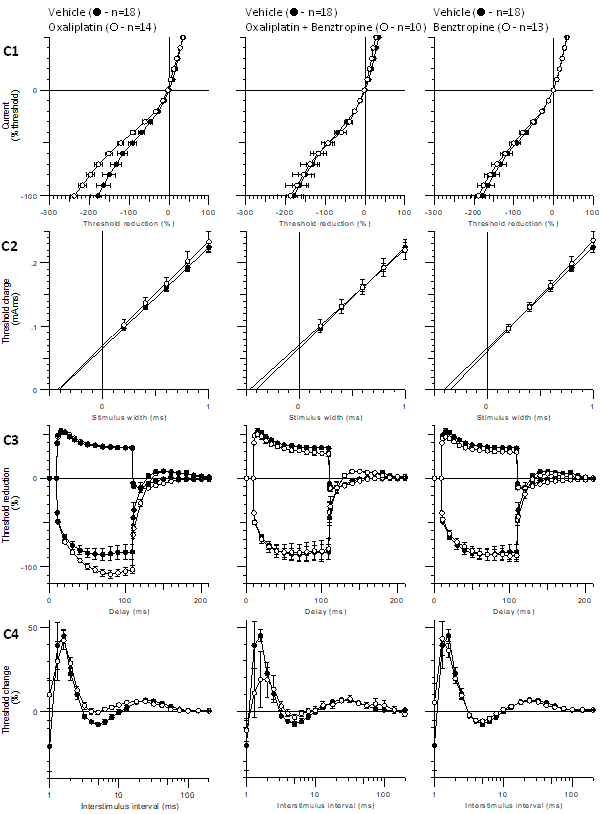

Supplement: Supplementary file 1 — Figure S1. Benztropine prevents the in vivo effects induced by oxaliplatin on mouse neuromuscular excitability curves. Excitability curves (means ± SD) were recorded at the plantar muscle in response to sciatic motor nerve stimulation of mice treated for 6 weeks with vehicle (black circles, n = 18), oxaliplatin (white circles, n = 14, left panels), oxaliplatin plus benztropine (white circles, n = 10, middle panels) or benztropine alone (white circles, n = 13, right panels). (C1) Current-threshold relationship [excitability modifications in response to depolarizing (up) and hyperpolarizing (down) currents], (C2) strength-duration relationship, (C3) threshold electrotonus in response to constant depolarizing (up) and hyperpolarizing (down) long-duration currents applied at sub-threshold intensity (± 40%), and (C4) recovery cycle. Note the absence of effect of oxaliplatin plus benztropine and benztropine alone, versus vehicle, on excitability waveforms. (TIF 1435 kb) [file 40478_2019_657_MOESM1_ESM.tif]

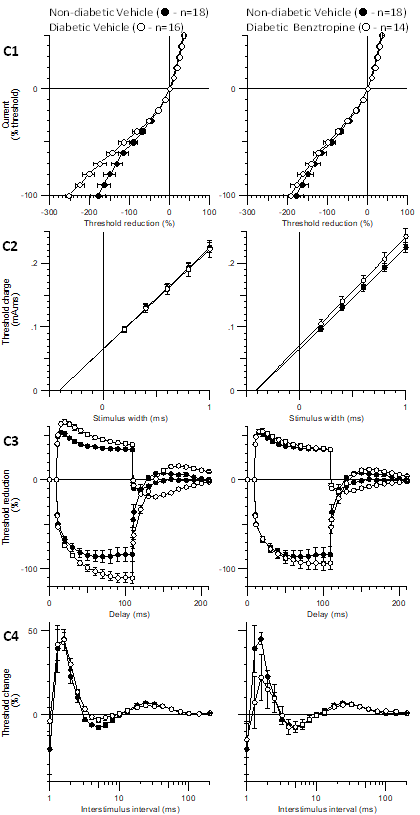

Supplement: Supplementary file 2 — Figure S2. Benztropine prevents the in vivo effects induced by diabetes on mouse neuromuscular excitability curves. Excitability curves (means ± SD) were recorded at the plantar muscle in response to sciatic motor nerve stimulation of mice treated for 6 weeks with non-diabetic vehicle (black circles, n = 18), diabetic vehicle (white circles, n = 16, left panels), and diabetic benztropine (white circles, n = 14, right panels). (C1) Current-threshold relationship [excitability modifications in response to depolarizing (up) and hyperpolarizing (down) currents], (C2) strength-duration relationship, (C3) threshold electrotonus in response to constant depolarizing (up) and hyperpolarizing (down) long-duration currents applied at sub-threshold intensity (± 40%), and (C4) recovery cycle. Note the absence of effect of diabetic benztropine, versus non-diabetic vehicle, on excitability waveforms. (TIF 1006 kb) [file 40478_2019_657_MOESM2_ESM.tif]

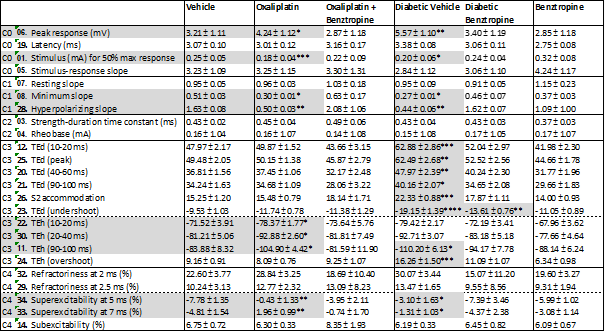

Supplement: Supplementary file 3 — : Table S1. Benztropine prevents the in vivo effects induced by oxaliplatin and diabetes on mouse neuromuscular excitability variables. Variables (means ± SD) derived from excitability curves established from plantar muscle recordings in response to sciatic motor nerve stimulation of mice treated with vehicle (n = 18), oxaliplatin (n = 14), oxaliplatin plus benztropine (n = 10), diabetic vehicle (n = 16), diabetic benztropine (n = 14) or benztropine alone (n = 13) for 6 weeks. (C0) Stimulus-response relationship, (C1) current-threshold relationship, (C2) strength-duration relationship, (C3) threshold electrotonus in response to constant depolarizing (TEd) and hyperpolarizing (TEh) long-duration currents applied at sub-threshold intensity (± 40%), and (C4) recovery cycle. *p < 0.05, **p < 0.01, ***p < 0.001, and ****p < 0.0001 versus vehicle (highlighted in grey). Note that, compared to vehicle; most if not all variables modified with oxaliplatin and diabetic vehicle remain unchanged with oxaliplatin plus benztropine, diabetic benztropine or benztropine alone. (TIF 593 kb) [file 40478_2019_657_MOESM3_ESM.tif]

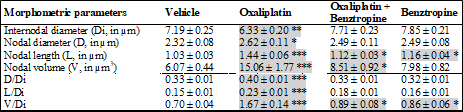

Supplement: Supplementary file 4 — : Table S2. Comparison of morphometric parameters. Mean values ± SD of morphometric parameters of single myelinated axons isolated from sciatic nerves of mice injected with vehicle, oxaliplatin, oxaliplatin plus benztropine or benztropine alone, for 6 weeks (137–206 axons from 4 different mice under each condition). *P = 0.011–0.049, **P = 0.006 and ***P < 0.0001 versus vehicle (highlighted in grey). (TIF 155 kb) [file 40478_2019_657_MOESM4_ESM.tif]
